# Serum lactate dehydrogenase is associated with impaired lung function: NHANES 2011–2012

**DOI:** 10.1371/journal.pone.0281203

**Published:** 2023-02-02

**Authors:** Sheng Hu, Jiayue Ye, Qiang Guo, Sheng Zou, Wenxiong Zhang, Deyuan Zhang, Yang Zhang, Silin Wang, Lang Su, Yiping Wei

**Affiliations:** Department of Thoracic Surgery, The Second Affiliated Hospital of Nanchang University, Nanchang, Jiangxi Province, P. R. China; Bolu Abant İzzet Baysal University: Bolu Abant Izzet Baysal Universitesi, TURKEY

## Abstract

**Background:**

Serum lactate dehydrogenase levels reflect disease status in a variety of organs, but its role in indicating pulmonary function is not yet clear. Therefore, this study explored the correlation between pulmonary function and serum lactate dehydrogenase, and investigated thresholds for changes in pulmonary function indicators in the total population as well as in different strata of the population.

**Methods:**

Based on data from the National Health and Nutrition Examination Survey (NHANES) 2011–2012 (n = 3453), univariate and stratified analyses were performed to investigate factors associated with pulmonary function, and multiple regression analysis was used to further investigate the specific relationship with serum lactate dehydrogenase. Smoothed curve fitting, threshold effect and saturation effect analysis were used to explore the threshold level of serum lactate dehydrogenase at the onset of changes in pulmonary function indicators.

**Results:**

Adjusted smoothed curve fit plots showed a linear relationship between serum lactate dehydrogenase levels and forced vital capacity and forced expiratory volume in one second: for each 1 U/L increase in serum lactate dehydrogenase levels, forced vital capacity decreased by 1.24 mL (95% CI = -2.05, -0.42, P = 0.0030) and forced expiratory volume in one second by 1.11 mL (95% CI = -1.82, -0.39, P = 0.0025).

**Conclusions:**

Serum lactate dehydrogenase was negatively and linearly correlated with pulmonary function indices in the total population analyzed. Based on the total population and different population stratifications, this study determined the threshold values of serum lactate dehydrogenase at the onset of decline of pulmonary function in different populations. This provides a new serological monitoring indicator for patients suffering from respiratory diseases and has implications for patients with possible clinical impairment of pulmonary function. However, our cross-sectional study was not able to determine a causal relationship between these two factors, and further research is needed.

## Introduction

The leading causes of disability and death worldwide are respiratory diseases, such as chronic obstructive pulmonary disease (COPD) and asthma, respiratory viral infections, and lung cancer. Since they have a mostly chronic progressive course they are a serious social and economic burden [1, 2]. Pulmonary function tests (PFTs) are used to assess the lung status of patients over time and have become an important component of pulmonary disease assessment programs [3]. Currently, the common metrics reported in PFTs are forced expiratory volume in one-second (FEV1) and the ratio to forced vital capacity (FVC). Because pulmonary function reflects the respiratory function of an individual, it is widely used for preoperative diagnosis of respiratory disease, surgical tolerance, postoperative assessment of patient recovery, and clinical management. In clinical practice, however, PFTs are contraindicated in patients with conditions such as severe cardiovascular disease, hemoptysis, active tuberculosis, poorly controlled hypertension, recent sinus surgery or middle ear surgery or infection, recent abdominal or thoracic surgery, or inability to follow instructions [4]. In addition, although PFTs are widely available in large hospitals, they remain to be improved in primary care hospitals due to uneven development [5]. This makes it difficult for clinicians to correctly assess the pulmonary function of patients and increases the risk of misdiagnosis and missed diagnosis.

Serological indicators may be a way to indirectly assess pulmonary function: previous studies found a significant correlation between serological indicator KL-6, cysteine-rich 61 and lung function tests in patients with respiratory diseases [6, 7]. Hence, developing universal serological screening indicators may be more accurate and efficient as well as less contraindicated. Serum lactate dehydrogenase (LDH) is an important oxidoreductase enzyme of the glycolytic pathway that is widely present in human tissues and usually elevated during inflammatory processes. Previous studies have found that LDH plays an important role as an indicator of inflammation in organ damage and is also commonly used in the diagnosis of myocardial infarction [8, 9], liver disease [10, 11], and malignancy [12–14]. The relationship between lactate dehydrogenase and pulmonary function in clinical practice is currently unclear although elevated concentrations of lactate dehydrogenase have been found in the serum of COPD patients and smoking patients [15–17]. Many of the previous study populations were not representative, which may have led to an underestimation of the clinical significance of lactate dehydrogenase.

The National Health and Nutrition Examination Survey (NHANES) is a multi-phase, ongoing, representative survey conducted by the CDC to assess the health status of the U.S. population based on a large body of data [18, 19]. The rigor and reliability of NHANES data has been confirmed by numerous studies [20, 21], so data from NHANES 2011–2012 were used in this study. Our goal was to conduct an in-depth and detailed stratified study to assess the relationship between LDH and pulmonary function indicators.

## Materials and methods

### Ethics statement

This study was approved by the ethical review committee of the National Center for Health Statistics (NCHS) and the ethical review committee of the Second Clinical School of Nanchang University. Written, informed consent was obtained from the participants.

### Study population

**Fig 1:**
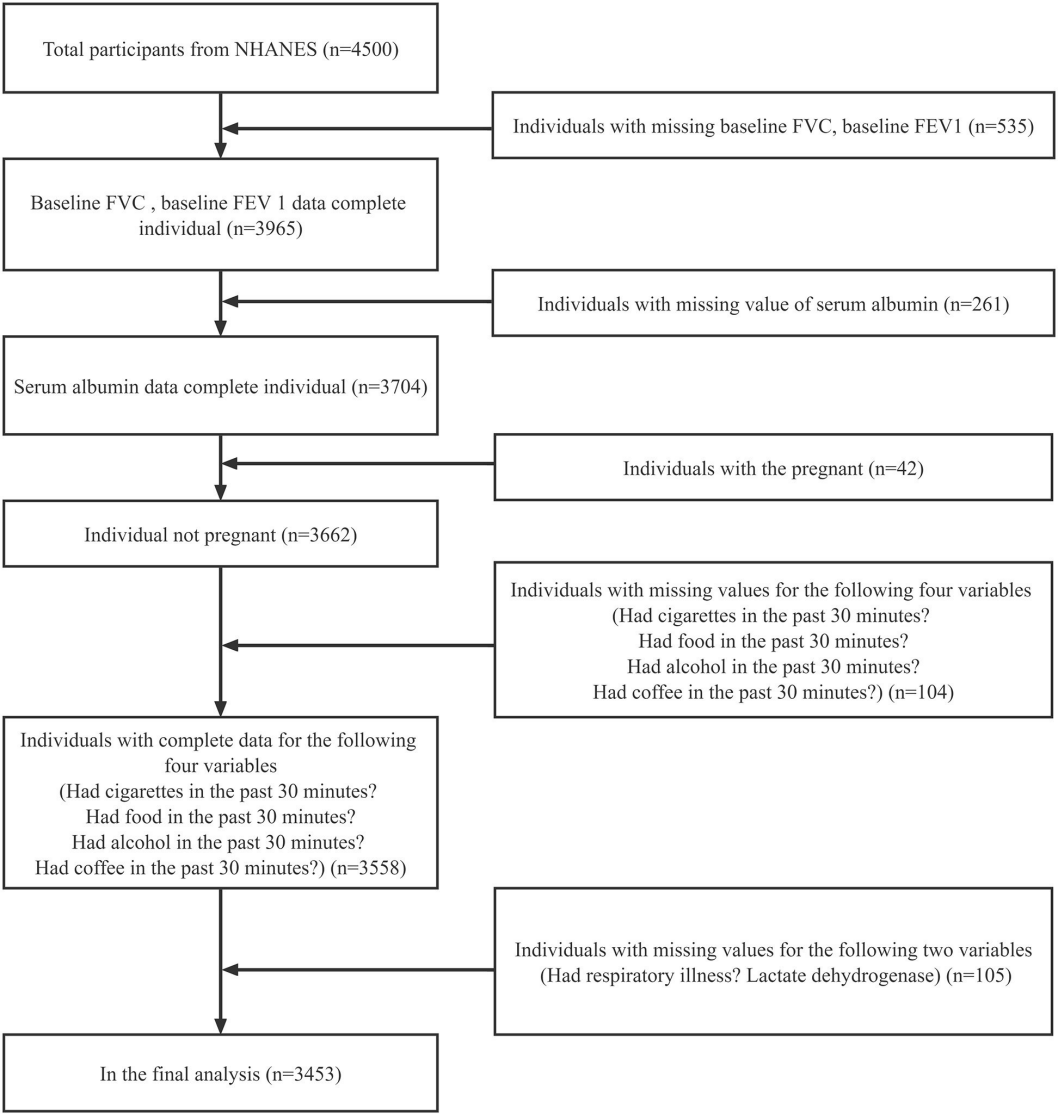
Flowchart of the screening process for selecting eligible participants from NHANES 2011–2012.

The data for this study were obtained from NHANES III, and detailed information on the survey methodology and data collection is available on the NCHS website (http://www.cdc.gov/nchs/). Our analysis was based on data recorded from 2011 to 2012, the most recent data available for the pulmonary function indicators FVC and FEV 1. A total of 4500 individuals were included in our study. During data collation we excluded individuals with missing data on FVC, FEV 1, serum albumin levels, and LDH levels. We also excluded patients whose behavior prior to data collection could interfere with the findings, such as those collected after smoking, eating, drinking alcohol, and thirty minutes after drinking coffee. Finally, patients with data missing from their medical records such as pregnancy, history of respiratory disease, and chest surgery were also excluded. The final total was 3453 participants and the detailed process is shown in Fig 1.

### Variables

LDH was the exposure variable in this study. We divided levels into three groups: low was ≥32 to 114 U/L (n = 1123); medium was ≥114 to 133 U/L (n = 1131), and high was ≥133 to ≤491 U/L (n = 1199). These groupings were predetermined based on previous studies that found an association between LDH and respiratory function [15, 20, 21]. The outcome variables were FVC and FEV 1, which were measured based on the latest American Thoracic Society standard procedure for functional spirometry assessment. The following continuous covariates were included: age, weight (kg), standing height (cm), systolic and diastolic blood pressure (mmHg) serum glucose (mmol/L), albumin (g/L), globulin (g/L), cholesterol (mmol/L), creatinine (μmol/L), and alanine aminotransferase (ALT, U/L). The following categorical variables were included as covariates: gender, race, smoking, education level, chest or abdominal surgery, and respiratory disease. LDH was measured using LD reagent (lactic acid as substrate) DxC800 (Beckman Instruments Inc, Brea, USA), which uses an enzyme rate method to measure LD activity in biological fluids. The system monitors the rate of change of absorbance at 340 nm over a fixed time interval, which is proportional to the activity of LD in the sample. More information on LDH, FVC, FEV 1 and covariate assays is detailed at https://www.cdc.gov/nchs/nhanes/.

### Statistical analysis

SPSS v.26 (IBM Corporation, Armonk, NY, USA) and Empower Stats (https://www.empowerstats.com, X&Y Solutions, Inc., Boston, MA) were used for statistical analysis of all data. P<0.05 indicates a statistically significant difference. The relationship between LDH levels and FVC and FEV1 was analyzed according to a weighted multivariate logistic regression model. The non-linear link between lactate dehydrogenase level and FVC and FEV 1 was addressed using smooth curve fitting and a generalized additive model. We used smooth curve fitting to examine whether the independent variable was partitioned into intervals. We applied segmented regression (also known as piece-wise regression) that used a separate line segment to fit each interval. A log-likelihood ratio test comparing a one-line (non-segmented) model to a segmented regression model was used to determine whether threshold exists (when p<0.05 was considered to apply to the segmented model). The inflection point that connected the segments was based on the model that gave maximum likelihood, and it was determined using a two-step recursive method. For the analysis of differences between groups, we used a weighted chi-square test for categorical data and a weighted linear regression model for continuous variables.

## Results

### Baseline characteristics of participants

People in the high lactate dehydrogenase (LDH) group were older and had higher body weight and blood pressure. The results showed that in the group with different lactate dehydrogenase levels, FVC and FEV 1 were the only variables that decreased with increasing lactate dehydrogenase levels (P<0.001). Variables with non-significant differences included gender, height, serum glucose level, and respiratory disease. Among the serological indices, globulin, cholesterol, creatinine, and ALT showed a gradual increase with lactate dehydrogenase (P<0.001). The rest of the variables with statistically significant differences are detailed in Table 1.

**Table 1:** Baseline characteristics of participants (N = 3453).

Lactate dehydrogenase (U/L) Tertile	Low(≥32 to 114)	Middle(≥114 to 133)	High(≥133 to ≤491)	*P*-value
**Age, mean±SD (years)**	40.14 ± 14.11	43.00 ± 14.14	46.35 ± 14.02	<0.001
**Weight (kg)**	78.74 ± 19.54	81.76 ± 20.98	85.36 ± 23.36	<0.001
**Standing Height (cm)**	168.98 ± 9.50	168.55 ± 10.19	168.10 ± 10.13	0.104
**Systolic blood pressure (mmHg)**	117.91 ± 14.74	120.24 ± 15.52	125.51 ± 18.24	<0.001
**Diastolic blood pressure (mmHg)**	70.86 ± 11.16	72.44 ± 11.34	73.91 ± 12.93	<0.001
**Glucose, serum (mmol/L)**	5.53 ± 2.17	5.53 ± 2.04	5.61 ± 2.04	0.520
**Albumin (g/L)**	43.18 ± 3.25	43.40 ± 3.19	42.91 ± 3.25	0.001
**Globulin (g/L)**	28.33 ± 4.39	28.86 ± 4.37	29.41 ± 4.77	<0.001
**Cholesterol (mmol/L)**	4.81 ± 0.95	4.99 ± 1.04	5.10 ± 1.12	<0.001
**Creatinine (umol/L)**	74.74 ± 19.45	76.74 ± 23.20	80.73 ± 36.86	<0.001
**Alanine aminotransferase ALT (U/L)**	20.81 ± 10.72	24.33 ± 14.26	30.69 ± 26.50	<0.001
**Lactate dehydrogenase (U/L)**	101.28 ± 9.58	122.84 ± 5.37	153.03 ± 22.72	<0.001
**Baseline FVC (mL)**	4108.70 ± 1026.93	3982.67 ± 1085.83	3776.14 ± 1064.77	<0.001
**Baseline FEV 1 (mL)**	3281.56 ± 862.37	3152.22 ± 880.56	2971.93 ± 879.05	<0.001
**Gender (%)**				0.400
Male	50.4	53.2	52.1	
Female	49.6	46.8	47.9	
**Race/Hispanic origin (%)**				<0.001
Mexican American	10.6	11.2	10.8	
Other Hispanic	10.2	11.8	8.3	
Non-Hispanic white	39.1	35.1	30.3	
Non-Hispanic black	20.2	24.3	34.9	
Other races—Including multi-racial	19.9	17.6	15.6	
**Education level (%)**				<0.001
Less than 9th grade	5	7.3	7.6	
9-11th grade	11.9	12	14.6	
High school graduate	17.8	19.2	22.3	
Some college or AA degree	33.9	32.3	31.4	
College graduate or above	31.3	29.3	24.2	
**Thoracic/abdominal surgery**				0.021
Yes	16.7	19.5	21.3	
No	83.3	80.5	78.7	
**Respiratory disease**				0.058
Yes	16.2	16.4	19.5	
No	83.8	83.6	80.5	
**Cigarette**				0.007
Yes	3.7	1.9	1.8	
No	96.3	98.1	98.2	

Note: continuous variables were presented as mean±SD; categorical variables were presented as n (%). FVC: forced vital capacity; FEV1: Forced expiratory volume in one second.

### Univariate and stratified analysis of the relationship between serum lactate dehydrogenase and pulmonary function

The reference group for each variable in the univariate analysis was the first group. There was a negative correlation between LDH levels and pulmonary function (Table 2, P<0.001). For the baseline FVC analysis, the beta value (CI) for LDH levels was -126.02 (-213.51, -38.53) in the middle tertile group and -332.56 (-418.80, -246.31) in the high tertile group compared to the low tertile group, both P<0.0001. For analysis of baseline FEV 1, the beta value (CI) of LDH levels was -129.34 (-201.51, -57.16) in the middle tertile group and -309.63 (-380.78, -238.48) in the high tertile group compared to the low tertile group, both P<0.0001. Age, gender, race, education level, thoracic/abdominal surgery, respiratory disease, weight, height, and systolic blood pressure were associated with FVC and FEV1 as detailed in Table 2 (P<0.05). For baseline FVC and FEV 1, differences in serum glucose and cholesterol were significant only in the higher tertile groups. Smoking was only significantly associated with FVC and not with FEV 1. Diastolic blood pressure was not significantly related to either FVC or FEV 1. Therefore, for further study, a stratified analysis was performed (S1 Table).

**Table 2:** Crude univariate analysis for baseline FVC and baseline FEV 1.

Exposure	Statistics	Baseline FVC (mL) β(95%CI) P	Baseline FEV 1 (mL) β(95%CI) P
**Lactate dehydrogenase (U/L)**	126.31 ± 25.96	-5.68 (-7.04, -4.32) <0.0001	-5.12 (-6.25, -4.00) <0.0001
**Lactate dehydrogenase (U/L) Tertile**			
Low	1123 (32.52%)	0	0
Middle	1131 (32.75%)	-126.02 (-213.51, -38.53) 0.0048	-129.34 (-201.51, -57.16) 0.0005
High	1199 (34.72%)	-332.56 (-418.80, -246.31) <0.0001	-309.63 (-380.78, -238.48) <0.0001
**Age (years)**	43.23 ± 14.31	-27.59 (-29.90, -25.27) <0.0001	-30.87 (-32.66, -29.09) <0.0001
**Age (years) Tertile**			
Low	1135 (32.87%)	0	0
Middle	1112 (32.20%)	-305.25 (-387.77, -222.72) <0.0001	-409.34 (-473.52, -345.16) <0.0001
High	1206 (34.93%)	-909.90 (-990.78, -829.01) <0.0001	-1017.19 (-1080.09, -954.29) <0.0001
**Gender**			
Male	1793 (51.93%)	0	0
Female	1660 (48.07%)	-1337.96 (-1393.59, -1282.33) <0.0001	-986.47 (-1035.40, -937.54) <0.0001
**Race/Hispanic origin**			
Mexican American	376 (10.89%)	0	0
Other Hispanic	347 (10.05%)	-246.87 (-394.76, -98.99) 0.0011	-182.61 (-307.22, -57.99) 0.0041
Non-Hispanic white	1199 (34.72%)	325.31 (207.89, 442.73) <0.0001	147.85 (48.91, 246.80) 0.0034
Non-Hispanic black	921 (26.67%)	-493.60 (-615.18, -372.02) <0.0001	-416.15 (-518.60, -313.70) <0.0001
Other races—Including multi-racial	610 (17.67%)	-307.81 (-438.06, -177.55) <0.0001	-200.60 (-310.36, -90.84) 0.0003
**Education level (%)**			
Less than 9th grade	229 (6.63%)	0	0
9-11th grade	445 (12.89%)	205.73 (36.09, 375.37) 0.0175	188.19 (48.15, 328.22) 0.0085
High school graduate	684 (19.81%)	264.61 (105.36, 423.86) 0.0011	238.23 (106.77, 369.69) 0.0004
Some college or AA degree	1122 (32.49%)	309.07 (157.82, 460.32) <0.0001	302.57 (177.72, 427.43) <0.0001
College graduate or above	973 (28.18%)	396.50 (243.30, 549.71) <0.0001	372.00 (245.53, 498.47) <0.0001
**Thoracic/abdominal surgery**			
Yes	663 (19.20%)	0	0
No	2790 (80.80%)	443.22 (353.96, 532.48) <0.0001	426.33 (352.89, 499.77) <0.0001
**Respiratory disease**			
Yes	601 (17.41%)	0	0
No	2852 (82.59%)	151.43 (57.58, 245.29) 0.0016	151.71 (74.16, 229.25) 0.0001
**Cigarette**			
Yes	85 (2.46%)	0	0
No	3368 (97.54%)	-316.59 (-546.33, -86.84) 0.0069	-177.82 (-367.87, 12.23) 0.0668
**Weight (kg)**	82.02 ± 21.56	10.47 (8.85, 12.09) <0.0001	7.22 (5.87, 8.57) <0.0001
**Weight (kg) Tertile**			
Low	1143 (33.27%)	0	0
Middle	1144 (33.29%)	465.97 (380.93, 551.01) <0.0001	309.38 (238.40, 380.36) <0.0001
High	1149 (33.44%)	595.51 (510.57, 680.46) <0.0001	410.99 (340.09, 481.89) <0.0001
**Standing Height (cm)**	168.53 ± 9.95	78.59 (76.15, 81.03) <0.0001	58.60 (56.37, 60.82) <0.0001
**Standing Height (cm) Tertile**			
Low	1136 (33.05%)	0	0
Middle	1150 (33.46%)	784.13 (719.90, 848.36) <0.0001	572.14 (515.04, 629.23) <0.0001
High	1151 (33.49%)	1775.87 (1711.65, 1840.08) <0.0001	1327.41 (1270.33, 1384.49) <0.0001
**Systolic blood pressure (mmHg)**	121.31 ± 16.58	-7.56 (-9.73, -5.38) <0.0001	-8.76 (-10.55, -6.97) <0.0001
**Systolic blood pressure (mmHg) Tertile**			
Low	1050 (31.70%)	0	0
Middle	1102 (33.27%)	213.46 (124.15, 302.77) <0.0001	124.48 (50.91, 198.04) 0.0009
High	1160 (35.02%)	-137.59 (-225.80, -49.38) 0.0023	-229.84 (-302.50, -157.18) <0.0001
**Diastolic blood pressure (mmHg)**	72.44 ± 11.92	3.59 (0.54, 6.63) 0.0210	0.44 (-2.08, 2.97) 0.7298
**Diastolic blood pressure (mmHg) Tertile**			
Low	988 (29.83%)	0	0
Middle	1210 (36.53%)	66.21 (-23.38, 155.80) 0.1476	4.71 (-69.49, 78.91) 0.9010
High	1114 (33.64%)	84.63 (-6.69, 175.94) 0.0694	-17.95 (-93.57, 57.68) 0.6419
**Glucose, serum (mmol/L)**	5.56 ± 2.09	-60.91 (-77.88, -43.94) <0.0001	-58.41 (-72.40, -44.41) <0.0001
**Glucose, serum (mmol/L) Tertile**			
Low	1113 (32.23%)	0	0
Middle	1119 (32.41%)	-35.03 (-123.10, 53.05) 0.4358	-46.19 (-118.57, 26.20) 0.2112
High	1221 (35.36%)	-273.30 (-359.52, -187.08) <0.0001	-311.36 (-382.23, -240.50) <0.0001
**Albumin (g/L)**	43.16 ± 3.24	114.79 (104.47, 125.10) <0.0001	102.05 (93.62, 110.48) <0.0001
**Albumin (g/L) Tertile**			
Low	1027 (29.74%)	0	0
Middle	1257 (36.40%)	389.15 (305.67, 472.63) <0.0001	323.37 (255.00, 391.74) <0.0001
High	1169 (33.85%)	854.16 (769.28, 939.03) <0.0001	758.62 (689.11, 828.14) <0.0001
**Globulin (g/L)**	28.88 ± 4.54	-67.87 (-75.40, -60.34) <0.0001	-47.61 (-53.91, -41.31) <0.0001
**Globulin (g/L) Tertile**			
Low	1030 (29.88%)	0	0
Middle	1002 (29.07%)	-314.50 (-403.97, -225.03) <0.0001	-238.57 (-313.31, -163.83) <0.0001
High	1415 (41.05%)	-691.55 (-774.13, -608.96) <0.0001	-491.29 (-560.28, -422.30) <0.0001
**Cholesterol (mmol/L)**	4.97 ± 1.05	-107.05 (-140.91, -73.20) <0.0001	-111.59 (-139.49, -83.69) <0.0001
**Cholesterol (mmol/L) Tertile**			
Low	1130 (32.73%)	0	0
Middle	1163 (33.68%)	-43.84 (-130.96, 43.29) 0.3241	-56.88 (-128.69, 14.93) 0.1207
High	1160 (33.59%)	-224.22 (-311.40, -137.04) <0.0001	-246.06 (-317.92, -174.20) <0.0001
**Creatinine (umol/L)**	77.47 ± 27.87	6.47 (5.21, 7.73) <0.0001	4.38 (3.33, 5.42) <0.0001
**Creatinine (umol/L) Tertile**			
Low	1121 (32.46%)	0	0
Middle	1161 (33.62%)	602.70 (520.03, 685.37) <0.0001	434.11 (364.69, 503.52) <0.0001
High	1171 (33.91%)	853.80 (771.30, 936.30) <0.0001	609.69 (540.42, 678.96) <0.0001
**Alanine aminotransferase ALT (U/L)**	25.39 ± 19.09	7.24 (5.39, 9.09) <0.0001	5.01 (3.48, 6.55) <0.0001
**Alanine aminotransferase ALT (U/L) Tertile**			
Low	1140 (33.02%)	0	0
Middle	1112 (32.21%)	273.95 (187.55, 360.36) <0.0001	170.55 (98.69, 242.40) <0.0001
High	1200 (34.76%)	528.56 (443.77, 613.34) <0.0001	373.70 (303.19, 444.20) <0.0001

Note: continuous variables were presented as mean±SD; categorical variables were presented as n (%). The first group was used as the reference (β = 0) for each univariate analysis group; (a) including multi-Racial; (b) includes 12th grade with no diploma; (c) GED or equivalent. Weighted by: full sample mobile examination center exam weight. Abbreviations: FVC: forced vital capacity; FEV1, forced expiratory volume in one second.

### Multiple regression equation analysis of the relationship between serum lactate dehydrogenase levels and pulmonary function

The results of multivariate analysis showed a negative correlation between LDH and pulmonary function (Table 3, P<0.01). In the different models, the beta values of both FVC and FEV 1 decreased progressively with increasing lactate dehydrogenase levels. In the unadjusted model, lactate dehydrogenase levels were associated with lower FVC (β = -126.02, 95% CI = -213.51, -38.53, P<0.001) and FEV 1 (β = -129.34, 95% CI = -201.51, -57.16, P<0.0001) in the intermediate subgroup compared with the low tertile group. Higher subgroup lactate dehydrogenase levels were associated with lower FVC (β = -332.56, 95% CI = -418.80, -246.31, p<0.001) and FEV 1 (β = -309.63, 95% CI = -380.78, -238.48, p<0.0001) compared to the lower tertile group. In adjusted models I and II, high lactate dehydrogenase levels were also associated with lower FVC and FEV 1 (Table 3). In fully adjusted model III, high lactate dehydrogenase levels were associated with lower FVC (β = -56.75, 95% CI = -105.43, -8.08, p<0.05) and FEV 1 (β = -53.28, 95% CI = -95.95, -10.62, p<0.05). The covariates used for adjustment in the model are detailed in Table 3.

**Table 3:** Relationship between serum and serum lactate dehydrogenase and pulmonary function (multiple regression equation analysis).

Outcome	Rough model β (95%CI) P-value	Model I β (95%CI) P-value	Model II β (95%CI) P-value	Model III β (95%CI) P-value
**Y = Baseline FVC (mL)**				
**Lactate dehydrogenase (U/L)**	-5.68 (-7.04, -4.32) <0.0001	-3.65 (-4.61, -2.69) <0.0001	-2.52 (-3.41, -1.64) <0.0001	-1.24 (-2.05, -0.42) 0.0030
**Lactate dehydrogenase (U/L) Tertile**				
Low	0	0	0	0
Middle	-126.02 (-213.51, -38.53) 0.0048	-90.41 (-151.52, -29.29) 0.0038	-67.63 (-123.22, -12.03) 0.0172	-35.67 (-82.16, 10.82) 0.1328
High	-332.56 (-418.80, -246.31) <0.0001	-196.47 (-257.48, -135.47) <0.0001	-131.46 (-187.38, -75.53) <0.0001	-56.75 (-105.43, -8.08) 0.0224
**Y = Baseline FEV 1 (mL)**				
**Lactate dehydrogenase (U/L)**	-5.12 (-6.25, -4.00) <0.0001	-2.66 (-3.42, -1.89) <0.0001	-1.87 (-2.61, -1.14) <0.0001	-1.11 (-1.82, -0.39) 0.0025
**Lactate dehydrogenase (U/L) Tertile**				
Low	0	0	0	0
Middle	-129.34 (-201.51, -57.16) 0.0005	-72.77 (-121.66, -23.88) 0.0036	-58.56 (-104.81, -12.31) 0.0131	-43.40 (-84.15, -2.65) 0.0369
High	-309.63 (-380.78, -238.48) <0.0001	-143.64 (-192.45, -94.83) <0.0001	-98.52 (-145.05, -51.99) <0.0001	-53.28 (-95.95, -10.62) 0.0144

Abbreviations: FVC: forced vital capacity; FEV1: forced expiratory volume in one second. Weighted by: full sample mobile examination center exam weight. Outcome variable: baseline FVC; baseline FEV 1. Exposure variable: lactate dehydrogenase (U/L). Rough model: variables unadjusted. Model I adjusted by gender, age; Model II adjusted by: gender, age, race; Model Ⅲ adjusted by: age; gender; race/Hispanic origin; education level; thoracic/abdominal surgery (yes, no); respiratory disease (yes, no); cigarette (yes, no); weight; standing height; systolic blood pressure; diastolic blood pressure; glucose, serum; albumin; globulin; cholesterol; creatinine; alanine aminotransferase.

### Smooth curve fitting, threshold effect and saturation effect analysis between serum lactate dehydrogenase levels and pulmonary function

**Fig 2:**
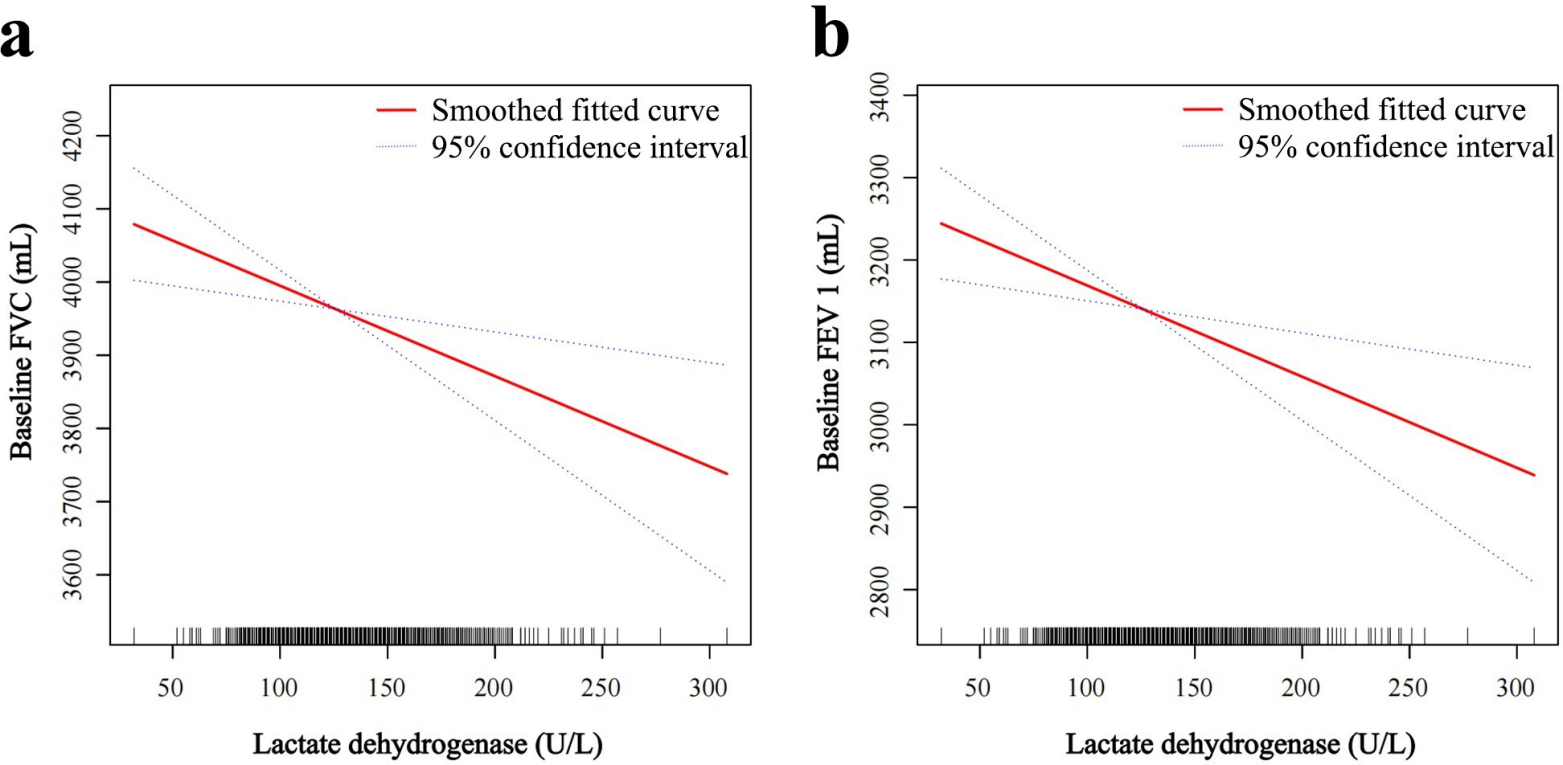
Association between serum lactate dehydrogenase and pulmonary function indicators FVC and FEV1. The red line represents the smoothed curve fit between the variables. (a) Solid line plots of curve fits for baseline lactate dehydrogenase and FVC for the main variables. (b) Solid line plots of curve fits for the primary variable between baseline lactate dehydrogenase and FEV 1. The blue line represents the 95% confidence interval of the fit. Full sample mobile examination center exam weight. Adjusted for age (smooth), sex, education, race, surgery (yes, no), respiratory disease (yes, no), cigarettes (yes, no), weight (smooth), standing height (smooth), diastolic blood pressure (smooth), systolic blood pressure (smooth), glucose, serum (smooth), cholesterol (smooth), creatinine (smooth), alanine aminotransferase (smooth), albumin (smooth), globulin (smooth).

To further clarify the relationship between LDH levels and lung function, we performed a smoothed curve fit (Fig 2) as well as threshold and saturation effect analyses (Table 4). The smoothed curve fit was adjusted to detect a nonlinear relationship, to determine the presence or absence of a threshold effect, and the feasibility of using linear regression. The results showed a linear relationship between LDH levels and FVC and FEV 1: for each 1 U/L increase in LDH levels, FVC decreased by 1.24 mL (95% CI = -2.05, -0.42, P = 0.0030) and FEV 1 decreased by 1.11 mL (95% CI = -1.82, -0.39, P = 0.0025; Fig 2A and 2B and Table 4). The covariates used for adjustment are detailed in Table 4.

**Table 4:** Analysis of threshold effect and saturation effect.

Outcome	Baseline FVC (mL) β (95%CI) P-value	Baseline FEV 1 (mL) β (95%CI) P-value
**Model I**		
A straight-line effect	-1.24 (-2.05, -0.42) 0.0030	-1.11 (-1.82, -0.39) 0.0025
**Model II**		
Fold points (K)	93	96
< K-segment effect 1	4.53 (-2.44, 11.50) 0.2027	0.86 (-4.36, 6.07) 0.7474
>K-segment Effect 2	-1.46 (-2.32, -0.60) 0.0009	-1.21 (-1.98, -0.44) 0.0020
Effect size difference of 2 versus 1	-5.99 (-13.18, 1.20) 0.1026	-2.07 (-7.50, 3.37) 0.4564
Equation predicted values at break points	4172.99 (4109.00, 4236.98)	3310.64 (3259.56, 3361.73)
Log likelihood ratio tests	0.101	0.455

Abbreviations: FVC: forced vital capacity; FEV1, forced expiratory volume in one second. Weighted by: full sample mobile examination center exam weight. Outcome variable: baseline FVC, baseline FEV 1. Exposure variable: lactate dehydrogenase. Adjusted for age, gender, race/Hispanic origin, education level, thoracic/abdominal surgery, respiratory disease, cigarette, weight, standing height, systolic blood pressure, diastolic blood pressure, glucose, serum, albumin, globulin, cholesterol, creatinine, alanine aminotransferase. When P<0.05 in Model I, the model showed a straight-line effect. When P>0.05 in Model I, the model showed a segmented effect in Model II, with the K value being the lactate dehydrogenase level at the fold point; β represents the slope of the curve, β for segments with P<0.05 was statistically significant. The K value is the inflection point, which is the level of lactate dehydrogenase content at which the relationship between lactate dehydrogenase and lung function changes.

### Smoothed curve fitting for each factor stratification, threshold effect and saturation effect analysis

**Fig 3:**
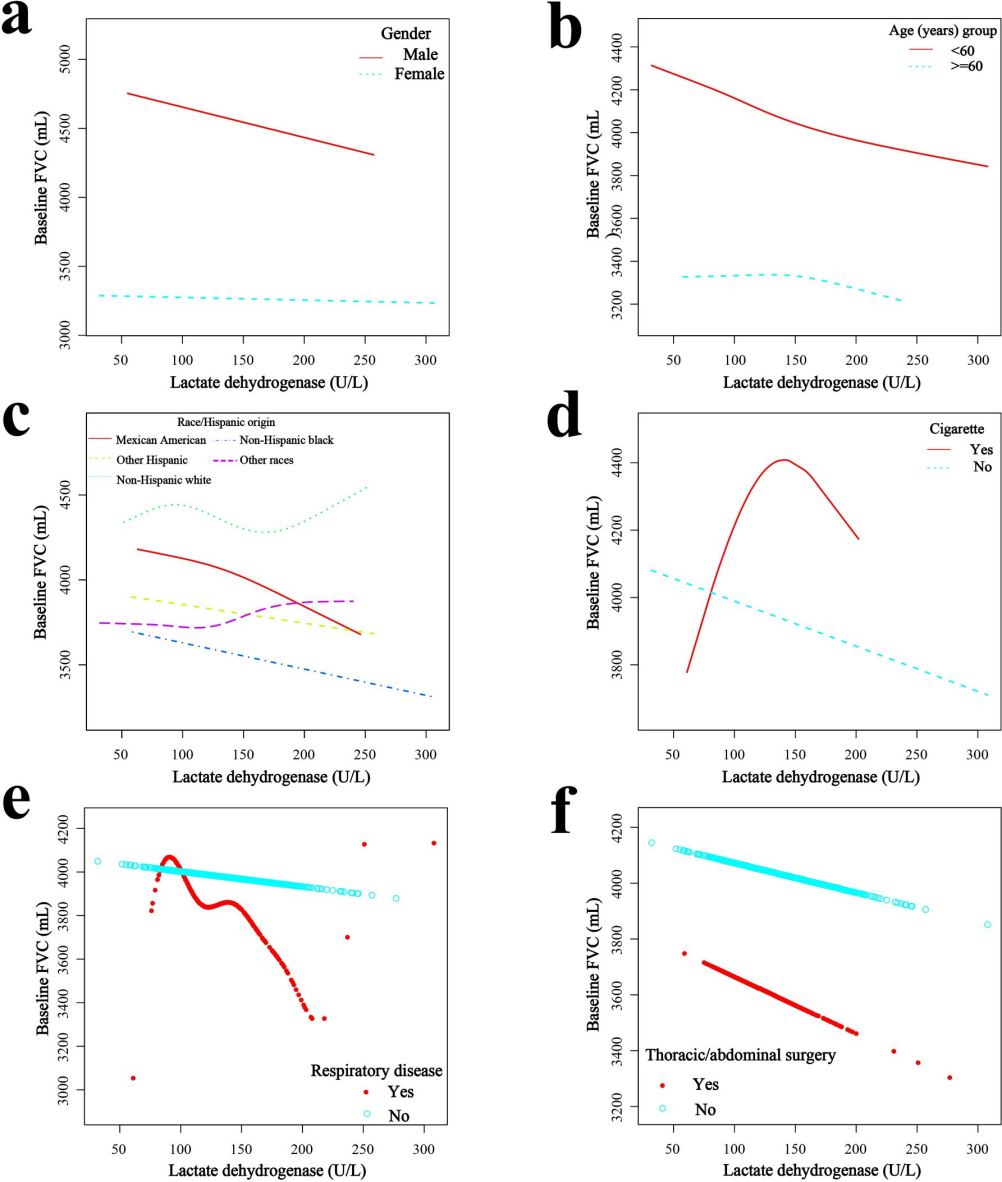
Relationship between serum lactate dehydrogenase and FVC. (a) Stratified by sex. (b) Stratified by age. (c) Stratified by race. (d) Stratified by smoking status. (e) Stratified by respiratory disease. (f) Stratified by surgery.

**Fig 4:**
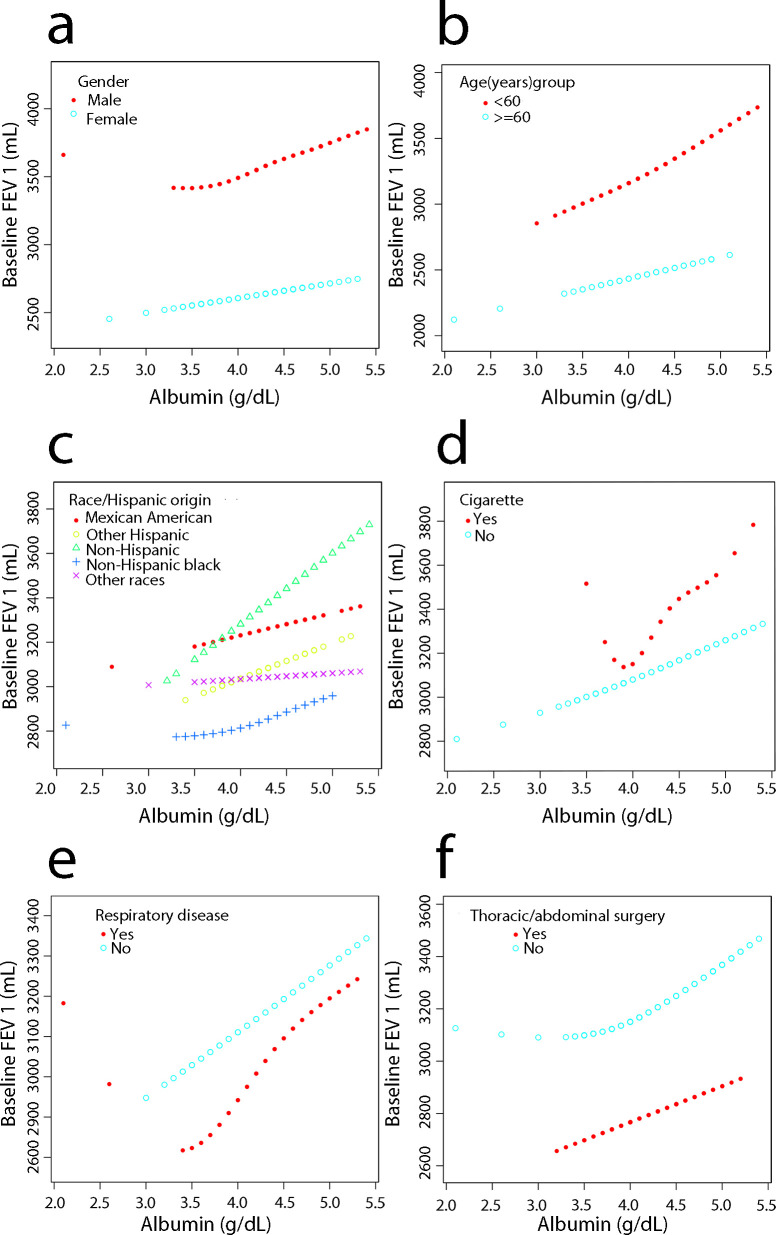
Relationship between serum albumin and FEV 1. (a) Stratified by sex. (b) Stratified by age. (c) Stratified by race. (d) Stratified by smoking status. (e) Stratified by respiratory disease. (f) Stratified by surgery.

Smooth-fit curves were plotted for the relationship between the different strata of the six covariates and LDH levels (Figs 3 and 4). For more detailed analysis, threshold effect and saturation effect analyses were performed to clarify the changes in FEV and FEV 1 with increasing LDH in the different strata of each covariate. A log-likelihood ratio of <0.05 in the table indicated that a segmented model was applicable. The k value is the turning point value, i.e., the level at which the relationship between LDH and lung function will probably change. Model II is not applicable when the relationship between LDH and the outcome variable shows a linear effect.

In men, LDH was linearly negatively correlated with both FVC (β = -1.57, 95% CI = -2.85 to -0.28, P = 0.017) and FEV1 (β = -1.75, 95% CI = -2.88 to -0.61, P = 0.026). In women, LDH was linearly and negatively correlated with FVC (β = -1.14, 95% CI = -2.09 to -0.18, P = 0.0202; Figs 3A and 4A and S2 Table). In people <60 years, LDH was linearly and negatively correlated with FVC (β = -2.07, 95% CI = -3.04 to -1.10, P<0.001). In addition, the relationship between LDH and FEV1 showed a segmental effect, with a negative correlation at levels >122 U/L (β = -4.14, 95% CI = -6.18 to -2.11, P<0.001). In those aged >60 years, the relationship between LDH and FVC showed a segmental effect with a negative correlation at LDH levels >163 U/L (β = -7.14, 95% CI = -13.14 to -0.81, P = 0.0275; Figs 3B and 4B and S3 Table).

There was a linear negative association with FVC among Mexican Americans (β = -2.98, 95% CI = -5.44 to -0.53, P = 0.0178) and non-Hispanic Black participants (β = -1.52, 95% CI = -2.82 to -0.23, P = 0.0216). LDH was negatively and linearly associated with FEV1 in Mexican Americans (β = -2.52, 95% CI = -4.42 to -0.62, P = 0.0098) and non-Hispanic Black participants (β = -1.21, 95% CI = -2.41 to -0.01, P = 0.0477). In non-Hispanic White participants (β = -1.21, 95% CI = -2.41 to -0.01, P = 0.0477), LDH had a segmental effect with FEV1, with a negative correlation when levels were <132 U/L (β = -3.44, 95% CI = -5.86 to -1.02, P = 0.0054; Figs 3C and 4C and S4 Table).

Among non-smokers, LDH was linearly and negatively correlated with both FVC (β = -1.33, 95% CI = -2.15 to -0.51, P = 0.0015) and FEV1 (β = -1.11, 95% CI = -1.83 to -0.39, P = 0.0026; Figs 3D and 4D and S5 Table).

In those with previous respiratory disease, LDH was linearly negatively associated with both FVC (β = -2.94, 95% CI = -4.86 to -1.03, P = 0.0028) and FEV 1 (β = -2.32, 95% CI = -4.11 to -0.52, P = 0.0116). In those without respiratory disease, serum albumin was linearly negatively correlated only with FEV 1 (β = -0.79, 95% CI = -1.57 to -0.01, P = 0.0471; Figs 3E and 4E and S6 Table).

In those without chest or abdominal surgery, LDH was linearly negatively correlated with both FVC (β = -1.03, 95% CI = -1.94 to -0.12, P = 0.0268) and FEV1 (β = -1.09, 95% CI = -1.89 to -0.29, P = 0.0075). In contrast, in those who had previous chest or abdominal surgery, LDH levels were linearly associated with FVC (β = -2.31, 95% CI = -4.14 to -0.49, P = 0.0134). There was a segmental effect with FEV1, which was negatively associated until levels were <113 U/L (β = -6.67, 95% CI = -12.03 to -1.31, P = 0.0150; Figs 3F and 4F and S7 Table).

## Discussion

Respiratory diseases remain a significant cause of morbidity and mortality worldwide. Ongoing research continues to improve diagnostic tools and treatment options [22–25], and current clinical investigations can be divided into laboratory and specific tests. Numerous previous studies have demonstrated the value of PFTs for clinical applications [26–28], such as in patients with COPD [29] and asthma [30]. However PFTs are not suitable for all patients [4]. For example, while they are not contraindicated in patients with tracheotomy or Morquio syndrome, performing PFTs is difficult and the results are not reliable [31, 32]. Furthermore, in the current phase of the COVID-19 epidemic, PFTs may be a potential route of transmission because of the aerosols generated during the procedure and the concentration of patients with pulmonary disease in the laboratory [33].

Serologic indicators are more universal than PFTs, have fewer contraindications, and can accurately and efficiently reflect relevant information about the sample. Previous studies have confirmed that more and more serologic markers are being used to diagnose and monitor diseases such as cancer, COVID-19, and cardiac diseases [34–38]. In addition, a large number of studies describe indirect associations between serologic indicators and pulmonary function [7, 15, 39], but the status of these indicators in the diagnosis and treatment of respiratory diseases needs to be further improved. The NHANES database has been used in many studies, and is a well-collected and representative population [40–43]. Hence, we obtained a large amount of valuable serological index data from this database for analysis and determined the potential value of LDH.

LDH, an important inflammatory marker, is underestimated in terms of its clinical significance [44]. Previous studies have suggested that LDH levels are associated with lung disease [45]. In recent years, it has not only been shown to be a prognostic marker for diseases such as non-small cell lung cancer [46], idiopathic pulmonary fibrosis [47], and metastatic breast cancer [48], but is also a common indicator in diagnosis [8–14]. In fact, LDH levels have important implications in pulmonary disease activity and response to therapy. Mura et al. plasma LDH was found to be induced by hypoxia and LDH levels were found to be increased in 22 patients diagnosed with IPF, but the relationship between LDH and IPF severity was unclear [49]. Spruit et al. showed that increased muscle LDH activity was found in older men with COPD and that resting serum LDH activity was increased in COPD patients compared to healthy smoking and non-smoking peers [50]. However, the relationship between LDH levels and pulmonary function was unclear. Previous studies have suggested that an inflammatory response due to impaired pulmonary function may be responsible for elevated levels [51]. LDH is present in cells, and when lung injury or inflammation decreases pulmonary function, LDH released from the cells increases serum levels. A previous study found lower indicators of pulmonary function and elevated serum LDH in patients with COPD relative to healthy patients [15]. Our results also showed a negative correlation between serum LDH and pulmonary function. Although serum LDH levels are not exactly equivalent to tissue LDH levels, tissue-level LDH expression may correlate with serum LDH levels [52]. Previous studies found that high expression of LDH in cancer mediates tumor immune escape leading to tumorigenesis or progression by suppressing the killing effect of immunity and promoting the suppressive effect of immunity. Thus, serum LDH may also indicate decreased pulmonary function due to progression of some respiratory cancers [53–56]. These studies provide guidance for future monitoring of serum LDH levels in response to changes in pulmonary function and predicting respiratory failure in specific populations.

Our study still has some limitations. The data from NHANES 2011–2012 are the most recent and representative data available that contain indicators of pulmonary function. In addition, although our sample size has improved compared to previous studies, data from a larger number of participants would have made the findings more convincing. Our cross-sectional study cannot mechanistically determine the causal relationship between these two factors, and further research is needed [57]. Although we controlled for confounding factors by statistical methods, we still may not be able to exclude the interference of other confounding factors. Hence, if more data are obtained or supported by more prospective and mechanistic studies, we believe that the relationship between LDH and pulmonary function will be more deeply interpreted in the future.

## Conclusions

The relationship between the serum marker lactate dehydrogenase and pulmonary function was explored in a large number of cases and in more detailed population stratification than previous studies. LDH levels were negatively correlated with pulmonary function. This study provides a new way to monitor changes in pulmonary function in patients for whom PFTs are clinically contraindicated. This provides a theoretical basis for lactate dehydrogenase as an indicator of pulmonary function. Identifying a threshold for LDH when PFTs begin to decline provides guidance for the diagnosis of respiratory disease.

## Supporting information

S1 Table Stratification analysis between serum lactate dehydrogenase and baseline FVC, serum lactate dehydrogenase and baseline FEV 1. (a) including Multi-Racial; (b) includes 12th grade with no diploma; (c) GED or equivalent. Weighted by: full sample mobile examination center exam weight.
(DOCX)

S2 Table Analysis of threshold effect and saturation effect (Stratification by gender). (DOCX)

S3 Table Analysis of threshold effect and saturation effect (Stratification by age). (DOCX)

S4 Table Analysis of threshold effect and saturation effect (Stratification by race/Hispanic origin). (DOCX)

S5 Table Analysis of threshold effect and saturation effect (Stratification by cigarette). (DOCX)

S6 Table Analysis of threshold effect and saturation effect (Stratification by respiratory disease). (DOCX)

S7 Table Analysis of threshold effect and saturation effect (Stratification by thoracic/abdominal surgery). (DOCX)

S1 Raw data (XLSX)

## Abbreviations

NHANES: national health and nutrition examination survey
COPD: chronic obstructive pulmonary disease
LDH: serum lactate dehydrogenase
FVC: forced vital capacity
FEV1: forced expiratory volume in the first second of expiration
PFTs: pulmonary function tests
NCHS: National Center for Health Statistics
CDC: centers for disease control and prevention
LD: lactic acid

## References

[1] The L. GBD 2017: a fragile world. Lancet. 2018;392(10159):1683. doi: 10.1016/S0140-6736(18)32858-7 30415747

[2] Fitzmaurice C, Abate D, Abbasi N, Abbastabar H, Abd-Allah F, Abdel-Rahman O, et al. Global, Regional, and National Cancer Incidence, Mortality, Years of Life Lost, Years Lived With Disability, and Disability-Adjusted Life-Years for 29 Cancer Groups, 1990 to 2017: A Systematic Analysis for the Global Burden of Disease Study. JAMA Oncol. 2019;5(12):1749–68. doi: 10.1001/jamaoncol.2019.2996 31560378 PMC6777271

[3] Drummond MB, McCormack MC. Integration of Pulmonary Function Data into Electronic Health Records: Time for Action. Am J Respir Crit Care Med. 2018;198(4):545–6. doi: 10.1164/rccm.201802-0378LE 29676933

[4] Coates AL, Graham BL, McFadden RG, McParland C, Moosa D, Provencher S, et al. Spirometry in primary care. Can Respir J. 2013;20(1):13–21. doi: 10.1155/2013/615281 23457669 PMC3628641

[5] Sheng W, Huang Y, Deng Z, Ma H. Investigation of the Prevalence and Diagnosis of Chronic Obstructive Pulmonary Disease in a Group of Elderly Individuals Residing in an Island Area of Ningbo. Can Respir J. 2019;2019:6918340. doi: 10.1155/2019/6918340 31467620 PMC6701409

[6] Jiang D, Xiao H, Dong R, Geng J, Xie B, Ren Y, et al. Krebs von den Lungen-6 levels in untreated idiopathic pulmonary fibrosis. Clin Respir J. 2022;16(3):234–43. doi: 10.1111/crj.13475 35081277 PMC9060088

[7] Tan ZX, Fu L, Wang WJ, Zhan P, Zhao H, Wang H, et al. Serum CYR61 Is Associated With Airway Inflammation and Is a Potential Biomarker for Severity in Chronic Obstructive Pulmonary Disease. Front Med (Lausanne). 2021;8:781596. doi: 10.3389/fmed.2021.781596 34917638 PMC8669148

[8] Miao P, Sheng S, Sun X, Liu J, Huang G. Lactate dehydrogenase A in cancer: a promising target for diagnosis and therapy. IUBMB Life. 2013;65(11):904–10. doi: 10.1002/iub.1216 24265197

[9] Halkin A, Stone GW, Grines CL, Cox DA, Rutherford BD, Esente P, et al. Prognostic implications of creatine kinase elevation after primary percutaneous coronary intervention for acute myocardial infarction. J Am Coll Cardiol. 2006;47(5):951–61. doi: 10.1016/j.jacc.2005.12.003 16516077

[10] Kounalakis N, Goydos JS. Tumor cell and circulating markers in melanoma: diagnosis, prognosis, and management. Curr Oncol Rep. 2005;7(5):377–82. doi: 10.1007/s11912-005-0065-2 16091200

[11] Tkaczewska J, Jamróz E, Piątkowska E, Borczak B, Kapusta-Duch J, Morawska M. Furcellaran-Coated Microcapsules as Carriers of Cyprinus carpio Skin-Derived Antioxidant Hydrolysate: An In Vitro and In Vivo Study. Nutrients. 2019;11(10). doi: 10.3390/nu11102502 31627407 PMC6835527

[12] Nemr S, Mayor-Modesto MH, Schwartz S, Summerhill EM. A 92-year-old woman with recurrent pleural effusions. Chest. 2008;134(1):196–9. doi: 10.1378/chest.07-2529 18628225

[13] Hinton JM. SERUM LACTATE DEHYDROGENASE IN BRONCHIAL CARCINOMA. Thorax. 1965;20(3):198–9. doi: 10.1136/thx.20.3.198 14292423 PMC1018920

[14] Denlinger CS, Cohen SJ. Progress in the development of prognostic and predictive markers for gastrointestinal malignancies. Curr Treat Options Oncol. 2007;8(5):339–51. doi: 10.1007/s11864-007-0045-x 18193357

[15] Fireman Klein E, Adir Y, Krencel A, Peri R, Vasserman B, Fireman E, et al. Ultrafine particles in airways: a novel marker of COPD exacerbation risk and inflammatory status. Int J Chron Obstruct Pulmon Dis. 2019;14:557–64. doi: 10.2147/COPD.S187560 30880945 PMC6402613

[16] He S, Zhang Q, Wu F, Chen J, He S, Ji Z, et al. Influence of cigarettes on myocardial injury in healthy population after exposure to high altitude over 5000 m. Sci Total Environ. 2023;855:158824. doi: 10.1016/j.scitotenv.2022.158824 36122711

[17] Zhang MY, Jiang YX, Yang YC, Liu JY, Huo C, Ji XL, et al. Cigarette smoke extract induces pyroptosis in human bronchial epithelial cells through the ROS/NLRP3/caspase-1 pathway. Life Sci. 2021;269:119090. doi: 10.1016/j.lfs.2021.119090 33465393

[18] Kenneson A, Funderburk JS. Patatin-like phospholipase domain-containing protein 3 (PNPLA3): A potential role in the association between liver disease and bipolar disorder. J Affect Disord. 2017;209:93–6. doi: 10.1016/j.jad.2016.11.035 27889599

[19] Zhang X, Bullard KM, Cotch MF, Wilson MR, Rovner BW, McGwin G Jr., et al. Association between depression and functional vision loss in persons 20 years of age or older in the United States, NHANES 2005–2008. JAMA Ophthalmol. 2013;131(5):573–81. doi: 10.1001/jamaophthalmol.2013.2597 23471505 PMC3772677

[20] Yang G, Sun T, Han YY, Rosser F, Forno E, Chen W, et al. Serum Cadmium and Lead, Current Wheeze, and Lung Function in a Nationwide Study of Adults in the United States. J Allergy Clin Immunol Pract. 2019;7(8):2653-60.e3. 10.1016/j.jaip.2019.05.029 PMC6842689 31146018

[21] Brigham EP, McCormack MC, Takemoto CM, Matsui EC. Iron status is associated with asthma and lung function in US women. PLoS One. 2015;10(2):e0117545. doi: 10.1371/journal.pone.0117545 25689633 PMC4331366

[22] Global, regional, and national deaths, prevalence, disability-adjusted life years, and years lived with disability for chronic obstructive pulmonary disease and asthma, 1990–2015: a systematic analysis for the Global Burden of Disease Study 2015. Lancet Respir Med. 2017;5(9):691–706. doi: 10.1016/S2213-2600(17)30293-X 28822787 PMC5573769

[23] Global, regional, and national life expectancy, all-cause mortality, and cause-specific mortality for 249 causes of death, 1980–2015: a systematic analysis for the Global Burden of Disease Study 2015. Lancet. 2016;388(10053):1459–544. doi: 10.1016/S0140-6736(16)31012-1 27733281 PMC5388903

[24] Pearce N, Aït-Khaled N, Beasley R, Mallol J, Keil U, Mitchell E, et al. Worldwide trends in the prevalence of asthma symptoms: phase III of the International Study of Asthma and Allergies in Childhood (ISAAC). Thorax. 2007;62(9):758–66. doi: 10.1136/thx.2006.070169 17504817 PMC2117323

[25] Burney PG, Patel J, Newson R, Minelli C, Naghavi M. Global and regional trends in COPD mortality, 1990–2010. Eur Respir J. 2015;45(5):1239–47. doi: 10.1183/09031936.00142414 25837037 PMC4531307

[26] Puente Maestú L, García de Pedro J. Lung function tests in clinical decision-making. Arch Bronconeumol. 2012;48(5):161–9. doi: 10.1016/j.arbres.2011.12.012 22364671

[27] Decramer M, Janssens W, Derom E, Joos G, Ninane V, Deman R, et al. Contribution of four common pulmonary function tests to diagnosis of patients with respiratory symptoms: a prospective cohort study. Lancet Respir Med. 2013;1(9):705–13. doi: 10.1016/S2213-2600(13)70184-X 24429274

[28] Sun LY, Gershon AS, Ko DT, Thilen SR, Yun L, Beattie WS, et al. Trends in Pulmonary Function Testing Before Noncardiothoracic Surgery. JAMA Intern Med. 2015;175(8):1410–2. doi: 10.1001/jamainternmed.2015.2087 26053615

[29] Neder JA, de-Torres JP, Milne KM, O’Donnell DE. Lung Function Testing in Chronic Obstructive Pulmonary Disease. Clin Chest Med. 2020;41(3):347–66. doi: 10.1016/j.ccm.2020.06.004 32800190

[30] Huang K, Yang T, Xu J, Yang L, Zhao J, Zhang X, et al. Prevalence, risk factors, and management of asthma in China: a national cross-sectional study. Lancet. 2019;394(10196):407–18. doi: 10.1016/S0140-6736(19)31147-X 31230828

[31] Sheshadri A, Keus L, Blanco D, Lei X, Kellner C, Shannon VR, et al. Pulmonary Function Testing in Patients with Tracheostomies: Feasibility and Technical Considerations. Lung. 2021;199(3):307–10. doi: 10.1007/s00408-021-00441-x 33779802 PMC9275556

[32] Kubaski F, Tomatsu S, Patel P, Shimada T, Xie L, Yasuda E, et al. Non-invasive pulmonary function test on Morquio patients. Mol Genet Metab. 2015;115(4):186–92. doi: 10.1016/j.ymgme.2015.06.007 26116954 PMC4706533

[33] Milanese M, Corsico AG, Bellofiore S, Carrozzi L, Di Marco F, Iovene B, et al. Suggestions for lung function testing in the context of COVID-19. Respir Med. 2020;177:106292. doi: 10.1016/j.rmed.2020.106292 33440299 PMC7773526

[34] Chung HW, Lee SG, Kim H, Hong DJ, Chung JB, Stroncek D, et al. Serum high mobility group box-1 (HMGB1) is closely associated with the clinical and pathologic features of gastric cancer. J Transl Med. 2009;7:38. doi: 10.1186/1479-5876-7-38 19476625 PMC2694170

[35] Li Y, Lai DY, Lei Q, Xu ZW, Wang F, Hou H, et al. Systematic evaluation of IgG responses to SARS-CoV-2 spike protein-derived peptides for monitoring COVID-19 patients. Cell Mol Immunol. 2021;18(3):621–31. doi: 10.1038/s41423-020-00612-5 33483707 PMC7821179

[36] Fuse K, Kodama M, Hanawa H, Okura Y, Ito M, Shiono T, et al. Enhanced expression and production of monocyte chemoattractant protein-1 in myocarditis. Clin Exp Immunol. 2001;124(3):346–52. doi: 10.1046/j.1365-2249.2001.01510.x 11472393 PMC1906075

[37] Alex P, Zachos NC, Nguyen T, Gonzales L, Chen TE, Conklin LS, et al. Distinct cytokine patterns identified from multiplex profiles of murine DSS and TNBS-induced colitis. Inflamm Bowel Dis. 2009;15(3):341–52. doi: 10.1002/ibd.20753 18942757 PMC2643312

[38] Zhao L, Yang Q, Liu J. Clinical Value Evaluation of microRNA-324-3p and Other Available Biomarkers in Patients With HBV Infection-Related Hepatocellular Carcinoma. Open Forum Infect Dis. 2021;8(6):ofab108. doi: 10.1093/ofid/ofab108 34189151 PMC8232384

[39] Shen Y, Yang T, Guo S, Li X, Chen L, Wang T, et al. Increased serum ox-LDL levels correlated with lung function, inflammation, and oxidative stress in COPD. Mediators Inflamm. 2013;2013:972347. doi: 10.1155/2013/972347 24078777 PMC3774040

[40] Wu WT, Li YJ, Feng AZ, Li L, Huang T, Xu AD, et al. Data mining in clinical big data: the frequently used databases, steps, and methodological models. Mil Med Res. 2021;8(1):44. doi: 10.1186/s40779-021-00338-z 34380547 PMC8356424

[41] Li Y, Pan A, Wang DD, Liu X, Dhana K, Franco OH, et al. Impact of Healthy Lifestyle Factors on Life Expectancies in the US Population. Circulation. 2018;138(4):345–55. doi: 10.1161/CIRCULATIONAHA.117.032047 29712712 PMC6207481

[42] Flegal KM, Carroll MD, Ogden CL, Curtin LR. Prevalence and trends in obesity among US adults, 1999–2008. Jama. 2010;303(3):235–41. doi: 10.1001/jama.2009.2014 20071471

[43] Egan BM, Zhao Y, Axon RN. US trends in prevalence, awareness, treatment, and control of hypertension, 1988–2008. Jama. 2010;303(20):2043–50. doi: 10.1001/jama.2010.650 20501926

[44] Zhou F, Yu T, Du R, Fan G, Liu Y, Liu Z, et al. Clinical course and risk factors for mortality of adult inpatients with COVID-19 in Wuhan, China: a retrospective cohort study. Lancet. 2020;395(10229):1054–62. doi: 10.1016/S0140-6736(20)30566-3 32171076 PMC7270627

[45] Drent M, Cobben NA, Henderson RF, Wouters EF, van Dieijen-Visser M. Usefulness of lactate dehydrogenase and its isoenzymes as indicators of lung damage or inflammation. Eur Respir J. 1996;9(8):1736–42. doi: 10.1183/09031936.96.09081736 8866602

[46] Loosen SH, Gorgulho J, Jördens MS, Schulze-Hagen M, Beier F, Vucur M, et al. Serum Levels of Soluble Urokinase Plasminogen Activator Receptor Predict Tumor Response and Outcome to Immune Checkpoint Inhibitor Therapy. Front Oncol. 2021;11:646883. doi: 10.3389/fonc.2021.646883 33869041 PMC8047604

[47] Kishaba T, Tamaki H, Shimaoka Y, Fukuyama H, Yamashiro S. Staging of acute exacerbation in patients with idiopathic pulmonary fibrosis. Lung. 2014;192(1):141–9. doi: 10.1007/s00408-013-9530-0 24221341

[48] Alarfi H, Youssef LA, Salamoon M. A Prospective, Randomized, Placebo-Controlled Study of a Combination of Simvastatin and Chemotherapy in Metastatic Breast Cancer. J Oncol. 2020;2020:4174395. doi: 10.1155/2020/4174395 32849871 PMC7436279

[49] Mura M, Belmonte G, Fanti S, Contini P, Pacilli AM, Fasano L, et al. Inflammatory activity is still present in the advanced stages of idiopathic pulmonary fibrosis. Respirology. 2005;10(5):609–14. doi: 10.1111/j.1440-1843.2005.00757.x 16268914

[50] Spruit MA, Pennings HJ, Does JD, Möller GM, Janssen PP, Wouters EF. Serum LDH and exercise capacity in COPD. Thorax. 2008;63(5):472. doi: 10.1136/thx.2007.086363 18443166

[51] Yan H, Liang X, Du J, He Z, Wang Y, Lyu M, et al. Proteomic and metabolomic investigation of serum lactate dehydrogenase elevation in COVID-19 patients. Proteomics. 2021;21(15):e2100002. doi: 10.1002/pmic.202100002 33987944 PMC8237019

[52] Koh YW, Lee SJ, Park SY. Prognostic significance of lactate dehydrogenase B according to histologic type of non-small-cell lung cancer and its association with serum lactate dehydrogenase. Pathol Res Pract. 2017;213(9):1134–8. doi: 10.1016/j.prp.2017.07.006 28756978

[53] Augoff K, Hryniewicz-Jankowska A, Tabola R. Lactate dehydrogenase 5: an old friend and a new hope in the war on cancer. Cancer Lett. 2015;358(1):1–7. doi: 10.1016/j.canlet.2014.12.035 25528630

[54] Feng Y, Xiong Y, Qiao T, Li X, Jia L, Han Y. Lactate dehydrogenase A: A key player in carcinogenesis and potential target in cancer therapy. Cancer Med. 2018;7(12):6124–36. doi: 10.1002/cam4.1820 30403008 PMC6308051

[55] Seth P, Csizmadia E, Hedblom A, Vuerich M, Xie H, Li M, et al. Deletion of Lactate Dehydrogenase-A in Myeloid Cells Triggers Antitumor Immunity. Cancer Res. 2017;77(13):3632–43. doi: 10.1158/0008-5472.CAN-16-2938 28446465 PMC5505499

[56] Ding J, Karp JE, Emadi A. Elevated lactate dehydrogenase (LDH) can be a marker of immune suppression in cancer: Interplay between hematologic and solid neoplastic clones and their microenvironments. Cancer Biomark. 2017;19(4):353–63. doi: 10.3233/CBM-160336 28582845 PMC13020749

[57] Marcinkevage JA, Alverson CJ, Narayan KM, Kahn HS, Ruben J, Correa A. Race/ethnicity disparities in dysglycemia among U.S. women of childbearing age found mainly in the nonoverweight/nonobese. Diabetes Care. 2013;36(10):3033–9. doi: 10.2337/dc12-2312 23780951 PMC3781530

