# Supplementary material for: Serum lactate dehydrogenase is associated with impaired lung function: NHANES 2011–2012
Source: PLoS One. 2023 Feb 2;18(2):e0281203. doi: 10.1371/journal.pone.0281203 (PMC9894433; doi:10.1371/journal.pone.0281203)
Supplement: S1 Table — (a) including Multi-Racial; (b) includes 12th grade with no diploma; (c) GED or equivalent. Weighted by: full sample mobile examination center exam weight. (DOCX) [file pone.0281203.s001.docx]

**S1 Table. Stratification analysis between serum lactate dehydrogenase and baseline FVC, serum lactate dehydrogenase and baseline FEV 1.**

| **Sub-group**  **X= Lactate dehydrogenase (U/L) Tertile** | **N** | **Baseline FVC (mL)**  **β (95%CI) P value** | **Baseline FEV 1 (mL)**  **β (95%CI) P value** |
| --- | --- | --- | --- |
| **Age (years) Tertile** |  |  |  |
| **Low** |  |  |  |
| Low | 467 | 0 | 0 |
| Middle | 376 | -11.90 (-149.59, 125.79) 0.8656 | -26.32 (-137.57, 84.92) 0.6429 |
| High | 292 | 60.70 (-87.55, 208.96) 0.4224 | 9.90 (-109.89, 129.68) 0.8714 |
| **Middle** |  |  |  |
| Low | 363 | 0 | 0 |
| Middle | 357 | -130.63 (-277.53, 16.27) 0.0816 | -98.77 (-212.86, 15.33) 0.0900 |
| High | 392 | -215.00 (-358.55, -71.44) 0.0034 | -152.45 (-263.95, -40.95) 0.0075 |
| **High** |  |  |  |
| Low | 293 | 0 | 0 |
| Middle | 398 | -44.38 (-189.17, 100.41) 0.5481 | -26.62 (-134.97, 81.73) 0.6303 |
| High | 515 | -356.38 (-494.02, -218.74) <0.0001 | -251.46 (-354.46, -148.46) <0.0001 |
| **Gender** |  |  |  |
| **Male** |  |  |  |
| Low | 566 | 0 | 0 |
| Middle | 602 | -42.23 (-149.53, 65.06) 0.4405 | -71.92 (-166.68, 22.84) 0.1370 |
| High | 625 | -264.38 (-370.72, -158.05) <0.0001 | -258.54 (-352.45, -164.63) <0.0001 |
| **Female** |  |  |  |
| Low | 557 | 0 | 0 |
| Middle | 529 | -293.60 (-373.57, -213.64) <0.0001 | -248.12 (-317.68, -178.55) <0.0001 |
| High | 574 | -449.88 (-528.22, -371.54) <0.0001 | -397.14 (-465.30, -328.98) <0.0001 |
| **Race/Hispanic origin** |  |  |  |
| **Mexican American** |  |  |  |
| Low | 119 | 0 | 0 |
| Middle | 127 | 41.00 (-192.88, 274.88) 0.7314 | 3.46 (-184.15, 191.08) 0.9712 |
| High | 130 | -118.72 (-351.29, 113.85) 0.3177 | -98.57 (-285.14, 87.99) 0.3011 |
| **Other Hispanic** |  |  |  |
| Low | 114 | 0 | 0 |
| Middle | 133 | -84.97 (-333.38, 163.44) 0.5030 | -77.22 (-288.32, 133.87) 0.4738 |
| High | 100 | -229.37 (-496.03, 37.28) 0.0927 | -196.97 (-423.57, 29.64) 0.0893 |
| **Non-Hispanic white** |  |  |  |
| Low | 439 | 0 | 0 |
| Middle | 397 | -108.82 (-256.61, 38.97) 0.1492 | -142.78 (-268.07, -17.49) 0.0257 |
| High | 363 | -329.59 (-480.97, -178.21) <0.0001 | -347.38 (-475.72, -219.05) <0.0001 |
| **Non-Hispanic black** |  |  |  |
| Low | 227 | 0 | 0 |

**Continued S1 Table.**

| **Sub-group**  **X= Lactate dehydrogenase (U/L) Tertile** | **N** | **Baseline FVC (mL)**  **β (95%CI) P value** | **Baseline FEV 1 (mL)**  **β (95%CI) P value** |
| --- | --- | --- | --- |
| Middle | 275 | -174.33 (-340.14, -8.51) 0.0396 | -127.79 (-268.94, 13.37) 0.0763 |
| High | 419 | -320.67 (-473.05, -168.28) <0.0001 | -259.86 (-389.58, -130.13) <0.0001 |
| **Other races^a^** |  |  |  |
| Low | 224 | 0 | 0 |
| Middle | 199 | -74.71 (-264.35, 114.92) 0.4403 | -102.84 (-259.51, 53.84) 0.1988 |
| High | 187 | -115.64 (-308.47, 77.20) 0.2403 | -182.39 (-341.70, -23.07) 0.0252 |
| **Education level** |  |  |  |
| **Less than 9th grade** |  |  |  |
| Low | 56 | 0 | 0 |
| Middle | 82 | -11.48 (-342.18, 319.22) 0.9458 | 3.71 (-256.99, 264.41) 0.9778 |
| High | 91 | -297.24 (-621.24, 26.75) 0.0735 | -262.92 (-518.34, -7.50) 0.0448 |
| **9-11th grade^b^** |  |  |  |
| Low | 134 | 0 | 0 |
| Middle | 136 | -51.36 (-295.59, 192.86) 0.6804 | -47.72 (-250.67, 155.23) 0.6451 |
| High | 175 | -373.69 (-604.02, -143.37) 0.0016 | -321.77 (-513.17, -130.37) 0.0011 |
| **High school graduate** |  |  |  |
| Low | 200 | 0 | 0 |
| Middle | 217 | -248.12 (-449.52, -46.73) 0.0160 | -188.51 (-361.31, -15.72) 0.0328 |
| High | 267 | -397.30 (-589.43, -205.16) <0.0001 | -303.52 (-468.37, -138.67) 0.0003 |
| **Some college or AA degree^c^** |  |  |  |
| Low | 381 | 0 | 0 |
| Middle | 365 | -178.68 (-331.96, -25.41) 0.0225 | -202.66 (-329.40, -75.92) 0.0018 |
| High | 376 | -273.89 (-426.01, -121.76) 0.0004 | -288.33 (-414.12, -162.54) <0.0001 |
| **College graduate or above** |  |  |  |
| Low | 352 | 0 | 0 |
| Middle | 331 | -20.34 (-182.86, 142.18) 0.8063 | -44.96 (-175.15, 85.22) 0.4986 |
| High | 290 | -287.42 (-455.76, -119.08) 0.0008 | -285.20 (-420.04, -150.36) <0.0001 |
| **Thoracic/abdominal surgery** |  |  |  |
| **Yes** |  |  |  |
| Low | 188 | 0 | 0 |
| Middle | 220 | -502.01 (-694.80, -309.22) <0.0001 | -359.97 (-511.01, -208.92) <0.0001 |
| High | 255 | -663.20 (-849.79, -476.60) <0.0001 | -472.90 (-619.09, -326.72) <0.000 |
| **No** |  |  |  |
| Low | 935 | 0 | 0 |
| Middle | 911 | -31.46 (-127.78, 64.87) 0.5222 | -65.84 (-145.80, 14.12) 0.1067 |
| High | 944 | -236.80 (-332.27, -141.33) <0.0001 | -252.19 (-331.45, -172.94) <0.0001 |
| **Respiratory disease** |  |  |  |
| **Yes** |  |  |  |
| Low | 182 | 0 | 0 |

**Continued S1 Table.**

| **Sub-group**  **X= Lactate dehydrogenase (U/L) Tertile** | **N** | **Baseline FVC (mL)**  **β (95%CI) P value** | **Baseline FEV 1 (mL)**  **β (95%CI) P value** |
| --- | --- | --- | --- |
| Middle | 185 | -287.70 (-507.52, -67.89) 0.0106 | -296.66 (-480.41, -112.92) 0.0016 |
| High | 234 | -459.34 (-667.43, -251.25) <0.0001 | -435.05 (-608.99, -261.11) <0.0001 |
| **No** |  |  |  |
| Low | 941 | 0 | 0 |
| Middle | 946 | -94.37 (-189.58, 0.83) 0.0521 | -96.58 (-174.81, -18.35) 0.0156 |
| High | 965 | -301.10 (-395.84, -206.37) <0.0001 | -278.50 (-356.35, -200.66) <0.0001 |
| **Cigarette** |  |  |  |
| **Yes** |  |  |  |
| Low | 41 | 0 | 0 |
| Middle | 22 | -7.07 (-556.96, 542.82) 0.9800 | -231.52 (-720.70, 257.67) 0.3563 |
| High | 22 | -15.98 (-565.87, 533.91) 0.9547 | -214.47 (-703.66, 274.72) 0.3927 |
| **No** |  |  |  |
| Low | 1082 | 0 | 0 |
| Middle | 1109 | -125.53 (-214.22, -36.84) 0.0056 | -124.80 (-197.87, -51.73) 0.0008 |
| High | 1177 | -335.44 (-422.85, -248.02) <0.0001 | -308.74 (-380.75, -236.72) <0.0001 |
| **Weight (kg) Tertile** |  |  |  |
| **Low** |  |  |  |
| Low | 427 | 0 | 0 |
| Middle | 378 | -251.36 (-366.46, -136.27) <0.0001 | -207.34 (-307.93, -106.75) <0.0001 |
| High | 338 | -370.54 (-489.19, -251.89) <0.0001 | -398.33 (-502.03, -294.63) <0.0001 |
| **Middle** |  |  |  |
| Low | 405 | 0 | 0 |
| Middle | 365 | -119.99 (-273.50, 33.51) 0.1258 | -155.63 (-280.88, -30.37) 0.0150 |
| High | 374 | -489.06 (-641.59, -336.53) <0.0001 | -438.31 (-562.77, -313.85) <0.0001 |
| **High** |  |  |  |
| Low | 289 | 0 | 0 |
| Middle | 380 | -74.28 (-247.11, 98.54) 0.3997 | -62.81 (-205.60, 79.98) 0.3888 |
| High | 480 | -322.14 (-487.00, -157.27) 0.0001 | -226.64 (-362.85, -90.43) 0.0011 |
| **Standing Height (cm) Tertile** |  |  |  |
| **Low** |  |  |  |
| Low | 344 | 0 | 0 |
| Middle | 388 | -202.01 (-293.08, -110.93) <0.0001 | -200.57 (-281.73, -119.41) <0.0001 |
| High | 404 | -334.59 (-424.81, -244.36) <0.0001 | -318.30 (-398.70, -237.90) <0.0001 |
| **Middle** |  |  |  |
| Low | 402 | 0 | 0 |
| Middle | 345 | -56.91 (-164.55, 50.72) 0.3002 | -45.27 (-141.72, 51.17) 0.3577 |
| High | 403 | -264.71 (-368.09, -161.33) <0.0001 | -255.37 (-348.00, -162.74) <0.0001 |
| **High** |  |  |  |
| Low | 374 | 0 | 0 |
| Middle | 392 | -63.46 (-193.36, 66.44) 0.3385 | -103.38 (-217.62, 10.86) 0.0764 |
| High | 385 | -289.82 (-420.29, -159.35) <0.0001 | -277.99 (-392.74, -163.25) <0.0001 |

**Continued S1 Table.**

| **Sub-group**  **X= Lactate dehydrogenase (U/L) Tertile** | **N** | **Baseline FVC (mL)**  **β (95%CI) P value** | **Baseline FEV 1 (mL)**  **β (95%CI) P value** |
| --- | --- | --- | --- |
| **Systolic blood pressure (mmHg) Tertile** |  |  |  |
| **Low** |  |  |  |
| Low | 419 | 0 | 0 |
| Middle | 368 | -93.68 (-227.58, 40.22) 0.1706 | -63.61 (-172.93, 45.70) 0.2543 |
| High | 263 | -226.92 (-374.36, -79.47) 0.0026 | -226.08 (-346.46, -105.71) 0.0002 |
| **Middle** |  |  |  |
| Low | 362 | 0 | 0 |
| Middle | 371 | -88.30 (-246.05, 69.44) 0.2728 | -129.44 (-259.18, 0.30) 0.0508 |
| High | 369 | -226.27 (-384.23, -68.32) 0.0051 | -245.14 (-375.06, -115.23) 0.0002 |
| **High** |  |  |  |
| Low | 298 | 0 | 0 |
| Middle | 347 | -211.83 (-380.72, -42.93) 0.0141 | -196.82 (-336.68, -56.97) 0.0059 |
| High | 515 | -437.53 (-593.18, -281.88) <0.0001 | -341.87 (-470.75, -212.98) <0.0001 |
| **Diastolic blood pressure (mmHg) Tertile** |  |  |  |
| **Low** |  |  |  |
| Low | 375 | 0 | 0 |
| Middle | 318 | -235.57 (-392.67, -78.47) 0.0034 | -193.76 (-326.83, -60.69) 0.0044 |
| High | 295 | -288.16 (-448.55, -127.78) 0.0004 | -281.95 (-417.79, -146.10) <0.0001 |
| **Middle** |  |  |  |
| Low | 415 | 0 | 0 |
| Middle | 396 | -101.29 (-248.72, 46.15) 0.1784 | -136.08 (-257.02, -15.14) 0.0276 |
| High | 399 | -371.05 (-518.20, -223.89) <0.0001 | -347.65 (-468.36, -226.94) <0.0001 |
| **High** |  |  |  |
| Low | 289 | 0 | 0 |
| Middle | 372 | -73.92 (-235.03, 87.19) 0.3687 | -65.99 (-197.45, 65.48) 0.3254 |
| High | 453 | -312.08 (-466.76, -157.40) <0.0001 | -266.05 (-392.27, -139.83) <0.0001 |
| **Glucose, serum (mmol/L) Tertile** |  |  |  |
| **Low** |  |  |  |
| Low | 401 | 0 | 0 |
| Middle | 358 | -94.75 (-241.42, 51.93) 0.2057 | -89.14 (-208.98, 30.71) 0.1452 |
| High | 354 | -368.17 (-515.28, -221.05) <0.0001 | -323.96 (-444.16, -203.76) <0.0001 |
| **Middle** |  |  |  |
| Low | 368 | 0 | 0 |
| Middle | 380 | -106.94 (-261.83, 47.96) 0.1763 | -135.60 (-263.68, -7.52) 0.0382 |
| High | 371 | -334.14 (-489.95, -178.32) <0.0001 | -319.76 (-448.61, -190.92) <0.0001 |
| **High** |  |  |  |
| Low | 354 | 0 | 0 |
| Middle | 393 | -146.45 (-297.49, 4.60) 0.0576 | -130.30 (-253.73, -6.87) 0.0388 |
| High | 474 | -250.32 (-395.12, -105.53) 0.0007 | -228.91 (-347.24, -110.58) 0.0002 |
| **Albumin (g/L) Tertile** |  |  |  |
| **Low** |  |  |  |

**Continued S1 Table.**

| **Sub-group**  **X= Lactate dehydrogenase (U/L) Tertile** | **N** | **Baseline FVC (mL)**  **β (95%CI) P value** | **Baseline FEV 1 (mL)**  **β (95%CI) P value** |
| --- | --- | --- | --- |
| Low | 333 | 0 | 0 |
| Middle | 311 | -85.86 (-225.87, 54.15) 0.2297 | -56.04 (-169.31, 57.22) 0.3324 |
| High | 383 | -277.46 (-410.49, -144.43) <0.0001 | -231.22 (-338.84, -123.60) <0.0001 |
| **Middle** |  |  |  |
| Low | 398 | 0 | 0 |
| Middle | 420 | -136.62 (-278.51, 5.27) 0.0594 | -155.79 (-270.30, -41.28) 0.0078 |
| High | 439 | -341.29 (-481.68, -200.90) <0.0001 | -297.73 (-411.03, -184.43) <0.0001 |
| **High** |  |  |  |
| Low | 392 | 0 | 0 |
| Middle | 400 | -177.94 (-324.87, -31.01) 0.0178 | -187.31 (-309.59, -65.03) 0.0027 |
| High | 377 | -297.10 (-446.24, -147.97) <0.0001 | -326.32 (-450.43, -202.21) <0.0001 |
| **Globulin (g/L) Tertile** |  |  |  |
| **Low** |  |  |  |
| Low | 391 | 0 | 0 |
| Middle | 327 | -51.14 (-212.22, 109.94) 0.5339 | -113.46 (-249.34, 22.42) 0.1020 |
| High | 312 | -323.10 (-486.27, -159.93) 0.0001 | -356.35 (-494.00, -218.70) <0.0001 |
| **Middle** |  |  |  |
| Low | 326 | 0 | 0 |
| Middle | 329 | -150.89 (-305.78, 3.99) 0.0565 | -121.66 (-250.66, 7.34) 0.0648 |
| High | 347 | -268.75 (-421.62, -115.87) 0.0006 | -235.38 (-362.71, -108.06) 0.0003 |
| **High** |  |  |  |
| Low | 405 | 0 | 0 |
| Middle | 474 | -60.29 (-189.46, 68.89) 0.3605 | -70.03 (-176.51, 36.45) 0.1976 |
| High | 536 | -250.57 (-376.26, -124.89) <0.0001 | -234.73 (-338.34, -131.13) <0.0001 |
| **Cholesterol (mmol/L) Tertile** |  |  |  |
| **Low** |  |  |  |
| Low | 420 | 0 | 0 |
| Middle | 356 | -116.76 (-271.02, 37.50) 0.1382 | -141.99 (-270.45, -13.53) 0.0305 |
| High | 354 | -280.61 (-435.11, -126.12) 0.0004 | -311.13 (-439.79, -182.48) <0.0001 |
| **Middle** |  |  |  |
| Low | 394 | 0 | 0 |
| Middle | 390 | -108.01 (-253.44, 37.41) 0.1457 | -84.77 (-206.49, 36.96) 0.1725 |
| High | 379 | -350.35 (-496.83, -203.87) <0.0001 | -309.58 (-432.18, -186.97) <0.0001 |
| **High** |  |  |  |
| Low | 309 | 0 | 0 |
| Middle | 385 | -117.16 (-272.88, 38.56) 0.1406 | -115.49 (-239.62, 8.64) 0.0685 |
| High | 466 | -304.33 (-453.90, -154.75) <0.0001 | -240.83 (-360.06, -121.60) <0.0001 |
| **Creatinine (umol/L) Tertile** |  |  |  |
| **Low** |  |  |  |
| Low | 419 | 0 | 0 |
| Middle | 358 | -227.91 (-343.58, -112.23) 0.0001 | -240.20 (-334.87, -145.53) <0.0001 |

**Continued S1 Table.**

| **Sub-group**  **X= Lactate dehydrogenase (U/L) Tertile** | **N** | **Baseline FVC (mL)**  **β (95%CI) P value** | **Baseline FEV 1 (mL)**  **β (95%CI) P value** |
| --- | --- | --- | --- |
| High | 344 | -294.16 (-411.09, -177.22) <0.0001 | -291.09 (-386.79, -195.39) <0.0001 |
| **Middle** |  |  |  |
| Low | 367 | 0 | 0 |
| Middle | 387 | -202.02 (-354.07, -49.97) 0.0093 | -191.87 (-318.38, -65.35) 0.0030 |
| High | 407 | -533.54 (-683.77, -383.32) <0.0001 | -485.06 (-610.05, -360.07) <0.0001 |
| **High** |  |  |  |
| Low | 337 | 0 | 0 |
| Middle | 386 | -90.82 (-246.77, 65.13) 0.2539 | -55.82 (-189.02, 77.37) 0.4116 |
| High | 448 | -372.89 (-523.73, -222.06) <0.0001 | -294.63 (-423.46, -165.80) <0.0001 |
| **Alanine aminotransferase ALT (U/L) Tertile** |  |  |  |
| **Low** |  |  |  |
| Low | 507 | 0 | 0 |
| Middle | 364 | -282.85 (-406.48, -159.23) <0.0001 | -231.86 (-337.02, -126.70) <0.0001 |
| High | 269 | -528.62 (-664.36, -392.88) <0.0001 | -468.79 (-584.26, -353.33) <0.0001 |
| **Middle** |  |  |  |
| Low | 359 | 0 | 0 |
| Middle | 371 | -204.28 (-358.05, -50.52) 0.0093 | -155.90 (-283.12, -28.69) 0.0165 |
| High | 382 | -518.28 (-670.95, -365.61) <0.0001 | -450.57 (-576.88, -324.26) <0.0001 |
| **High** |  |  |  |
| Low | 257 | 0 | 0 |
| Middle | 396 | -90.88 (-263.03, 81.27) 0.3010 | -148.17 (-289.12, -7.22) 0.0396 |
| High | 547 | -355.16 (-517.69, -192.64) <0.0001 | -322.65 (-455.73, -189.57) <0.0001 |

Note: (a) including Multi-Racial; (b) includes 12th grade with no diploma; (c) GED or equivalent. Weighted by: full sample mobile examination center exam weight.
