# Supplementary material for: Serum lactate dehydrogenase is associated with impaired lung function: NHANES 2011–2012
Source: PLoS One. 2023 Feb 2;18(2):e0281203. doi: 10.1371/journal.pone.0281203 (PMC9894433; doi:10.1371/journal.pone.0281203)
Supplement: S2 Table — (DOCX) [file pone.0281203.s002.DOCX]

**S2 Table. Analysis of threshold effect and saturation effect (Stratification by gender).**

| **Baseline FVC** | **Gender** | **Male**  **β(95%CI) *P*-value** | **Female**  **β(95%CI) *P*-value** | **Total**  **β(95%CI) *P*-value** |
| --- | --- | --- | --- | --- |
|  | **Model I** |  |  | P-interaction: 0.009 |
|  | A straight-line effect | -1.57 (-2.85, -0.28) 0.0170 | -1.14 (-2.09, -0.18) 0.0202 | -1.24 (-2.05, -0.42) 0.0030 |
|  | **Model II** |  |  | P-interaction: 0.039 |
|  | Fold points (K) | 99 | 93 | 93 |
|  | < K-segment effect 1 | 3.22 (-5.73, 12.17) 0.4806 | 4.38 (-2.66, 11.42) 0.2232 | 4.53 (-2.44, 11.50) 0.2027 |
|  | >K-segment Effect 2 | -1.87 (-3.28, -0.47) 0.0090 | -1.40 (-2.41, -0.39) 0.0069 | -1.46 (-2.32, -0.60) 0.0009 |
|  | Effect size difference of 2 versus 1 | -5.10 (-14.52, 4.33) 0.2893 | -5.78 (-13.09, 1.53) 0.1215 | -5.99 (-13.18, 1.20) 0.1026 |
|  | Equation predicted values at break points | 4756.86 (4682.20, 4831.51) | 3521.77 (3463.60, 3579.93) | 4172.99 (4109.00, 4236.98) |
|  | Log likelihood ratio tests | 0.286 | 0.118 | 0.101 |
| **Baseline FEV 1** | **Gender** | **Male**  **β(95%CI) *P*-value** | **Female**  **β(95%CI) *P*-value** | **Total**  **β(95%CI) *P*-value** |
|  | **Model I** |  |  | P-interaction: <0.001 |
|  | A straight-line effect | -1.75 (-2.88, -0.61) 0.0026 | -0.86 (-1.69, -0.04) 0.0411 | -1.11 (-1.82, -0.39) 0.0025 |
|  | **Model II** |  |  | P-interaction: 0.001 |
|  | Fold points (K) | 97 | 93 | 96 |
|  | < K-segment effect 1 | 1.05 (-7.87, 9.97) 0.8176 | 1.94 (-4.13, 8.00) 0.5314 | 0.86 (-4.36, 6.07) 0.7474 |
|  | >K-segment Effect 2 | -1.89 (-3.11, -0.67) 0.0025 | -0.99 (-1.87, -0.12) 0.0258 | -1.21 (-1.98, -0.44) 0.0020 |
|  | Effect size difference of 2 versus 1 | -2.94 (-12.24, 6.36) 0.5357 | -2.93 (-9.23, 3.37) 0.3617 | -2.07 (-7.50, 3.37) 0.4564 |
|  | Equation predicted values at break points | 3751.82 (3684.51, 3819.12) | 2857.32 (2806.66, 2907.98) | 3310.64 (3259.56, 3361.73) |
|  | Log likelihood ratio tests | 0.533 | 0.358 | 0.455 |

Abbreviations: FVC: forced vital capacity; FEV1: forced expiratory volume in one second. Outcome variable: baseline FVC (mL); baseline FEV 1 (mL); Exposure variable: lactate dehydrogenase. Adjusted for Age (years); Race/Hispanic origin; Education level; Thoracic/abdominal surgery; respiratory disease; cigarette; weight (kg); standing height (cm); systolic blood pressure (mmHg); diastolic blood pressure (mmHg); glucose, serum (mmol/L); albumin (g/L); globulin (g/L); cholesterol (mmol/L); creatinine (umol/L); alanine aminotransferase (U/L). When P<0.05 in Model I, the model showed a straight-line effect. When P>0.05 in Model I, the model showed a segmented effect in Model II, with the K value being the lactate dehydrogenase level at the fold point; β represents the slope of the curve, β for segments with P<0.05 was statistically significant. The K value is the inflection point, which is the level of lactate dehydrogenase content at which the relationship between lactate dehydrogenase and lung function changes.
