# Supplementary material for: Serum lactate dehydrogenase is associated with impaired lung function: NHANES 2011–2012
Source: PLoS One. 2023 Feb 2;18(2):e0281203. doi: 10.1371/journal.pone.0281203 (PMC9894433; doi:10.1371/journal.pone.0281203)
Supplement: S3 Table — (DOCX) [file pone.0281203.s003.DOCX]

**S3 Table. Analysis of threshold effect and saturation effect (Stratification by age).**

| **Baseline FVC** | **Age (years) group** | **<60**  **β(95%CI) *P*-value** | **>=60**  **β(95%CI) *P*-value** | **Total**  **β(95%CI) *P*-value** |
| --- | --- | --- | --- | --- |
|  | **Model I** |  |  | P-interaction: 0.085 |
|  | A straight-line effect | -2.07 (-3.04, -1.10) <0.0001 | -0.64 (-2.47, 1.20) 0.4974 | -1.76 (-2.61, -0.91) <0.0001 |
|  | **Model II** |  |  | P-interaction: 0.124 |
|  | Fold points (K) | 93 | 163 | 93 |
|  | < K-segment effect 1 | 4.59 (-3.57, 12.76) 0.2704 | 0.81 (-1.46, 3.08) 0.4839 | 3.06 (-4.24, 10.36) 0.4111 |
|  | >K-segment Effect 2 | -2.33 (-3.35, -1.31) <0.0001 | -7.14 (-13.47, -0.81) 0.0275 | -1.95 (-2.84, -1.05) <0.0001 |
|  | Effect size difference of 2 versus 1 | -6.93 (-15.36, 1.50) 0.1074 | -7.95 (-15.36, -0.54) 0.0359 | -5.01 (-12.53, 2.52) 0.1923 |
|  | Equation predicted values at break points | 4241.20 (4172.93, 4309.46) | 3141.14 (2986.41, 3295.88) | 4172.99 (4109.00, 4236.98) |
|  | Log likelihood ratio tests | 0.106 | 0.032 | 0.191 |
| **Baseline FEV 1** | **Age (years) group** | **<60**  **β(95%CI) *P*-value** | **>=60**  **β(95%CI) *P*-value** | **Total**  **β(95%CI) *P*-value** |
|  | **Model I** |  |  | P-interaction: 0.013 |
|  | A straight-line effect | -2.12 (-3.01, -1.22) <0.0001 | -0.59 (-2.24, 1.06) 0.4841 | -1.80 (-2.59, -1.01) <0.0001 |
|  | **Model II** |  |  | P-interaction: 0.023 |
|  | Fold points (K) | 122 | 163 | 121 |
|  | < K-segment effect 1 | -4.14 (-6.18, -2.11) <0.0001 | 0.56 (-1.49, 2.60) 0.5955 | -3.63 (-5.53, -1.72) 0.0002 |
|  | >K-segment Effect 2 | -1.08 (-2.37, 0.22) 0.1034 | -5.74 (-11.44, -0.03) 0.0493 | -0.96 (-2.08, 0.16) 0.0927 |
|  | Effect size difference of 2 versus 1 | 3.07 (0.30, 5.83) 0.0298 | -6.29 (-12.97, 0.39) 0.0654 | 2.67 (0.14, 5.20) 0.0390 |
|  | Equation predicted values at break points | 3270.36 (3220.66, 3320.06) | 2346.24 (2231.18, 2461.30) | 3153.28 (3106.65, 3199.91) |
|  | Log likelihood ratio tests | 0.029 | 0.060 | 0.038 |

Abbreviations: FVC: forced vital capacity; FEV1, forced expiratory volume in one second. Weighted by: full sample mobile examination center exam weight. Outcome variable: baseline FVC, baseline FEV 1. Exposure variable: lactate dehydrogenase. Adjusted for age, gender, race/Hispanic origin, education level, thoracic/abdominal surgery, respiratory disease, cigarette, weight, standing height, systolic blood pressure, diastolic blood pressure, glucose, serum, albumin, globulin, cholesterol, creatinine, alanine aminotransferase. When P<0.05 in Model I, the model showed a straight-line effect. When P>0.05 in Model I, the model showed a segmented effect in Model II, with the K value being the lactate dehydrogenase level at the fold point; β represents the slope of the curve, β for segments with P<0.05 was statistically significant. The K value is the inflection point, which is the level of lactate dehydrogenase content at which the relationship between lactate dehydrogenase and lung function changes.
