# Supplementary material for: Serum lactate dehydrogenase is associated with impaired lung function: NHANES 2011–2012
Source: PLoS One. 2023 Feb 2;18(2):e0281203. doi: 10.1371/journal.pone.0281203 (PMC9894433; doi:10.1371/journal.pone.0281203)
Supplement: S4 Table — (DOCX) [file pone.0281203.s004.DOCX]

**S4 Table. Analysis of threshold effect and saturation effect (Stratification by Race/Hispanic origin).**

| **Baseline FVC** | **Race/Hispanic origin** | **Mexican American**  **β(95%CI) *P*-value** | **Other Hispanic**  **β(95%CI) *P*-value** | **Non-Hispanic white**  **β(95%CI) *P*-value** | **Non-Hispanic black**  **β(95%CI) *P*-value** | **Other races - Including multi-racial**  **β(95%CI) *P*-value** | **Total**  **β(95%CI) *P*-value** |
| --- | --- | --- | --- | --- | --- | --- | --- |
|  | **Model I** |  |  |  |  |  | P-interaction: 0.069 |
|  | A straight-line effect | -2.98 (-5.44, -0.53) 0.0178 | -1.67 (-4.42, 1.08) 0.2344 | -1.69 (-3.30, -0.08) 0.0400 | -1.52 (-2.82, -0.23) 0.0216 | 1.52 (-0.47, 3.50) 0.1351 | -1.24 (-2.05, -0.42) 0.0030 |
|  | **Model II** |  |  |  |  |  | P-interaction: 0.223 |
|  | Fold points (K) | 134 | 111 | 93 | 153 | 122 | 93 |
|  | < K-segment effect 1 | -0.08 (-3.92, 3.76) 0.9661 | -3.00 (-11.44, 5.44) 0.4865 | 8.77 (-3.32, 20.86) 0.1554 | -0.96 (-2.97, 1.05) 0.3511 | -1.06 (-5.14, 3.03) 0.6118 | 4.53 (-2.44, 11.50) 0.2027 |
|  | >K-segment Effect 2 | -6.60 (-11.02, -2.17) 0.0037 | -1.28 (-4.90, 2.35) 0.4904 | -2.16 (-3.85, -0.46) 0.0128 | -2.42 (-5.21, 0.36) 0.0884 | 3.24 (0.13, 6.35) 0.0414 | -1.46 (-2.32, -0.60) 0.0009 |
|  | Effect size difference of 2 versus 1 | -6.51 (-13.16, 0.14) 0.0558 | 1.72 (-8.61, 12.06) 0.7439 | -10.92 (-23.44, 1.59) 0.0875 | -1.47 (-5.47, 2.54) 0.4726 | 4.30 (-1.66, 10.26) 0.1581 | -5.99 (-13.18, 1.20) 0.1026 |
|  | Equation predicted values at break points | 4132.41 (3974.37, 4290.44) | 3874.51 (3708.24, 4040.77) | 4567.88 (4456.26, 4679.50) | 3453.23 (3337.31, 3569.16) | 3670.07 (3543.01, 3797.12) | 4172.99 (4109.00, 4236.98) |
|  | Log likelihood ratio tests | 0.048 | 0.735 | 0.084 | 0.467 | 0.150 | 0.101 |

**Continued S4 Table.**

| **Baseline FEV 1** | **Race/Hispanic origin** | **Mexican American**  **β(95%CI) *P*-value** | **Other Hispanic**  **β(95%CI) *P*-value** | **Non-Hispanic white**  **β(95%CI) *P*-value** | **Non-Hispanic black**  **β(95%CI) *P*-value** | **Other races - Including multi-racial**  **β(95%CI) *P*-value** | **Total**  **β(95%CI) *P*-value** |
| --- | --- | --- | --- | --- | --- | --- | --- |
|  | **Model I** |  |  |  |  |  | P-interaction: 0.198 |
|  | A straight-line effect | -2.52 (-4.42, -0.62) 0.0098 | -1.26 (-3.57, 1.04) 0.2839 | -1.53 (-3.01, -0.04) 0.0438 | -1.21 (-2.41, -0.01) 0.0477 | 0.78 (-0.76, 2.32) 0.3201 | -1.11 (-1.82, -0.39) 0.0025 |
|  | **Model II** |  |  |  |  |  | P-interaction: 0.469 |
|  | Fold points (K) | 135 | 125 | 132 | 139 | 111 | 96 |
|  | < K-segment effect 1 | -1.56 (-4.49, 1.38) 0.2996 | 0.29 (-4.09, 4.66) 0.8985 | -3.44 (-5.86, -1.02) 0.0054 | 0.35 (-1.99, 2.69) 0.7726 | -3.03 (-7.54, 1.48) 0.1881 | 0.86 (-4.36, 6.07) 0.7474 |
|  | >K-segment Effect 2 | -3.79 (-7.30, -0.27) 0.0354 | -2.54 (-6.38, 1.30) 0.1954 | 0.84 (-1.96, 3.63) 0.5566 | -2.47 (-4.49, -0.45) 0.0168 | 1.95 (-0.06, 3.96) 0.0580 | -1.21 (-1.98, -0.44) 0.0020 |
|  | Effect size difference of 2 versus 1 | -2.23 (-7.42, 2.96) 0.4009 | -2.83 (-9.61, 3.96) 0.4149 | 4.28 (-0.01, 8.56) 0.0506 | -2.81 (-6.45, 0.82) 0.1300 | 4.98 (-0.56, 10.52) 0.0784 | -2.07 (-7.50, 3.37) 0.4564 |
|  | Equation predicted values at break points | 3280.30 (3152.77, 3407.83) | 3089.71 (2945.90, 3233.52) | 3288.43 (3197.99, 3378.86) | 2817.98 (2729.52, 2906.43) | 3035.53 (2931.52, 3139.54) | 3310.64 (3259.56, 3361.73) |
|  | Log likelihood ratio tests | 0.385 | 0.398 | 0.048 | 0.125 | 0.073 | 0.455 |

Abbreviations: FVC: forced vital capacity; FEV1, forced expiratory volume in one second. Weighted by: full sample mobile examination center exam weight. Outcome variable: baseline FVC, baseline FEV 1. Exposure variable: lactate dehydrogenase. Adjusted for age, gender, race/Hispanic origin, education level, thoracic/abdominal surgery, respiratory disease, cigarette, weight, standing height, systolic blood pressure, diastolic blood pressure, glucose, serum, albumin, globulin, cholesterol, creatinine, alanine aminotransferase. When P<0.05 in Model I, the model showed a straight-line effect. When P>0.05 in Model I, the model showed a segmented effect in Model II, with the K value being the lactate dehydrogenase level at the fold point; β represents the slope of the curve, β for segments with P<0.05 was statistically significant. The K value is the inflection point, which is the level of lactate dehydrogenase content at which the relationship between lactate dehydrogenase and lung function changes.
