# Supplementary material for: Serum lactate dehydrogenase is associated with impaired lung function: NHANES 2011–2012
Source: PLoS One. 2023 Feb 2;18(2):e0281203. doi: 10.1371/journal.pone.0281203 (PMC9894433; doi:10.1371/journal.pone.0281203)
Supplement: S5 Table — (DOCX) [file pone.0281203.s005.DOCX]

**S5 Table. Analysis of threshold effect and saturation effect (Stratification by Cigarette).**

| **Baseline FVC** | **Cigarette** | **Yes**  **β(95%CI) *P*-value** | **No**  **β(95%CI) *P*-value** | **Total**  **β(95%CI) *P*-value** |
| --- | --- | --- | --- | --- |
|  | **Model I** |  |  | P-interaction: 0.065 |
|  | A straight-line effect | 5.70 (-1.56, 12.96) 0.1295 | -1.33 (-2.15, -0.51) 0.0015 | -1.24 (-2.05, -0.42) 0.0030 |
|  | **Model II** |  |  | P-interaction: 0.083 |
|  | Fold points (K) | 158 | 93 | 93 |
|  | < K-segment effect 1 | 8.66 (0.27, 17.04) 0.0477 | 3.06 (-4.06, 10.17) 0.3996 | 4.53 (-2.44, 11.50) 0.2027 |
|  | >K-segment Effect 2 | -16.35 (-49.03, 16.33) 0.3309 | -1.50 (-2.36, -0.64) 0.0007 | -1.46 (-2.32, -0.60) 0.0009 |
|  | Effect size difference of 2 versus 1 | -25.01 (-61.17, 11.15) 0.1806 | -4.56 (-11.89, 2.78) 0.2234 | -5.99 (-13.18, 1.20) 0.1026 |
|  | Equation predicted values at break points | 4127.75 (3606.92, 4648.58) | 4162.34 (4097.52, 4227.17) | 4172.99 (4109.00, 4236.98) |
|  | Log likelihood ratio tests | 0.107 | 0.222 | 0.101 |
| **Baseline FEV 1** | **Cigarette** | **Yes**  **β(95%CI) *P*-value** | **No**  **β(95%CI) *P*-value** | **Total**  **β(95%CI) *P*-value** |
|  | **Model I** |  |  | P-interaction: 0.832 |
|  | A straight-line effect | -0.01 (-6.60, 6.59) 0.9983 | -1.11 (-1.83, -0.39) 0.0026 | -1.11 (-1.82, -0.39) 0.0025 |
|  | **Model II** |  |  | P-interaction: 0.373 |
|  | Fold points (K) | 100 | 120 | 96 |
|  | < K-segment effect 1 | 18.84 (-9.21, 46.88) 0.1933 | -1.80 (-3.61, 0.01) 0.0509 | 0.86 (-4.36, 6.07) 0.7474 |
|  | >K-segment Effect 2 | -2.91 (-10.70, 4.87) 0.4663 | -0.82 (-1.82, 0.19) 0.1103 | -1.21 (-1.98, -0.44) 0.0020 |
|  | Effect size difference of 2 versus 1 | -21.75 (-53.22, 9.72) 0.1809 | 0.98 (-1.37, 3.34) 0.4140 | -2.07 (-7.50, 3.37) 0.4564 |
|  | Equation predicted values at break points | 3518.53 (3186.73, 3850.33) | 3157.96 (3110.92, 3205.00) | 3310.64 (3259.56, 3361.73) |
|  | Log likelihood ratio tests | 0.107 | 0.412 | 0.455 |

Abbreviations: FVC: forced vital capacity; FEV1, forced expiratory volume in one second. Weighted by: full sample mobile examination center exam weight. Outcome variable: baseline FVC, baseline FEV 1. Exposure variable: lactate dehydrogenase. Adjusted for age, gender, race/Hispanic origin, education level, thoracic/abdominal surgery, respiratory disease, cigarette, weight, standing height, systolic blood pressure, diastolic blood pressure, glucose, serum, albumin, globulin, cholesterol, creatinine, alanine aminotransferase. When P<0.05 in Model I, the model showed a straight-line effect. When P>0.05 in Model I, the model showed a segmented effect in Model II, with the K value being the lactate dehydrogenase level at the fold point; β represents the slope of the curve, β for segments with P<0.05 was statistically significant. The K value is the inflection point, which is the level of lactate dehydrogenase content at which the relationship between lactate dehydrogenase and lung function changes.
