# Supplementary material for: Serum lactate dehydrogenase is associated with impaired lung function: NHANES 2011–2012
Source: PLoS One. 2023 Feb 2;18(2):e0281203. doi: 10.1371/journal.pone.0281203 (PMC9894433; doi:10.1371/journal.pone.0281203)
Supplement: S6 Table — (DOCX) [file pone.0281203.s006.DOCX]

**S6 Table. Analysis of threshold effect and saturation effect (Stratification by Respiratory disease).**

| **Bseeline FVC** | **Respiratory disease** | **Yes**  **β(95%CI) *P*-value** | **No**  **β(95%CI) *P*-value** | **Total**  **β(95%CI) *P*-value** |
| --- | --- | --- | --- | --- |
|  | **Model I** |  |  | P-interaction: 0.006 |
|  | A straight-line effect | -2.94 (-4.86, -1.03) 0.0028 | -0.79 (-1.69, 0.12) 0.0883 | -1.24 (-2.05, -0.42) 0.0030 |
|  | **Model II** |  |  | P-interaction: 0.012 |
|  | Fold points (K) | 93 | 93 | 93 |
|  | < K-segment effect 1 | 16.48 (-5.14, 38.10) 0.1357 | 2.65 (-4.69, 9.98) 0.4792 | 4.53 (-2.44, 11.50) 0.2027 |
|  | >K-segment Effect 2 | -3.48 (-5.48, -1.47) 0.0007 | -0.93 (-1.88, 0.02) 0.0561 | -1.46 (-2.32, -0.60) 0.0009 |
|  | Effect size difference of 2 versus 1 | -19.96 (-42.08, 2.16) 0.0776 | -3.58 (-11.16, 4.01) 0.3552 | -5.99 (-13.18, 1.20) 0.1026 |
|  | Equation predicted values at break points | 4154.18 (3998.98, 4309.38) | 4170.80 (4100.43, 4241.17) | 4172.99 (4109.00, 4236.98) |
|  | Log likelihood ratio tests | 0.071 | 0.353 | 0.101 |
| **Baseline FEV 1** | **Respiratory disease** | **Yes**  **β(95%CI) *P*-value** | **No**  **β(95%CI) *P*-value** | **Total**  **β(95%CI) *P*-value** |
|  | **Model I** |  |  | P-interaction: 0.032 |
|  | A straight-line effect | -2.32 (-4.11, -0.52) 0.0116 | -0.79 (-1.57, -0.01) 0.0471 | -1.11 (-1.82, -0.39) 0.0025 |
|  | **Model II** |  |  | P-interaction: 0.100 |
|  | Fold points (K) | 120 | 157 | 96 |
|  | < K-segment effect 1 | -5.47 (-10.37, -0.56) 0.0293 | -0.55 (-1.54, 0.44) 0.2749 | 0.86 (-4.36, 6.07) 0.7474 |
|  | >K-segment Effect 2 | -1.24 (-3.62, 1.13) 0.3065 | -1.65 (-3.96, 0.65) 0.1600 | -1.21 (-1.98, -0.44) 0.0020 |
|  | Effect size difference of 2 versus 1 | 4.23 (-1.90, 10.35) 0.1768 | -1.10 (-3.88, 1.67) 0.4362 | -2.07 (-7.50, 3.37) 0.4564 |
|  | Equation predicted values at break points | 2995.03 (2880.82, 3109.25) | 2982.57 (2913.67, 3051.47) | 3310.64 (3259.56, 3361.73) |
|  | Log likelihood ratio tests | 0.167 | 0.434 | 0.455 |

Abbreviations: FVC: forced vital capacity; FEV1, forced expiratory volume in one second. Weighted by: full sample mobile examination center exam weight. Outcome variable: baseline FVC, baseline FEV 1. Exposure variable: lactate dehydrogenase. Adjusted for age, gender, race/Hispanic origin, education level, thoracic/abdominal surgery, respiratory disease, cigarette, weight, standing height, systolic blood pressure, diastolic blood pressure, glucose, serum, albumin, globulin, cholesterol, creatinine, alanine aminotransferase. When P<0.05 in Model I, the model showed a straight-line effect. When P>0.05 in Model I, the model showed a segmented effect in Model II, with the K value being the lactate dehydrogenase level at the fold point; β represents the slope of the curve, β for segments with P<0.05 was statistically significant. The K value is the inflection point, which is the level of lactate dehydrogenase content at which the relationship between lactate dehydrogenase and lung function changes.
