# Supplementary material for: Serum lactate dehydrogenase is associated with impaired lung function: NHANES 2011–2012
Source: PLoS One. 2023 Feb 2;18(2):e0281203. doi: 10.1371/journal.pone.0281203 (PMC9894433; doi:10.1371/journal.pone.0281203)
Supplement: S7 Table — (DOCX) [file pone.0281203.s007.DOCX]

**S7 Table. Analysis of threshold effect and saturation effect (Stratification by Thoracic/abdominal surgery).**

| **Baseline FVC** | **Thoracic/abdominal surgery** | **Yes**  **β(95%CI) *P*-value** | **No**  **β(95%CI) *P*-value** | **Total**  **β(95%CI) *P*-value** |
| --- | --- | --- | --- | --- |
|  | **Model I** |  |  | P-interaction: 0.332 |
|  | A straight-line effect | -2.31 (-4.14, -0.49) 0.0134 | -1.03 (-1.94, -0.12) 0.0268 | -1.24 (-2.05, -0.42) 0.0030 |
|  | **Model II** |  |  | P-interaction: 0.316 |
|  | Fold points (K) | 115 | 93 | 93 |
|  | < K-segment effect 1 | -7.47 (-13.05, -1.89) 0.0089 | 6.00 (-1.52, 13.51) 0.1179 | 4.53 (-2.44, 11.50) 0.2027 |
|  | >K-segment Effect 2 | -0.85 (-3.21, 1.52) 0.4830 | -1.31 (-2.27, -0.35) 0.0074 | -1.46 (-2.32, -0.60) 0.0009 |
|  | Effect size difference of 2 versus 1 | 6.63 (-0.15, 13.40) 0.0557 | -7.31 (-15.07, 0.45) 0.0650 | -5.99 (-13.18, 1.20) 0.1026 |
|  | Equation predicted values at break points | 3658.61 (3535.28, 3781.95) | 4215.46 (4145.30, 4285.63) | 4172.99 (4109.00, 4236.98) |
|  | Log likelihood ratio tests | 0.051 | 0.064 | 0.101 |
| **Baseline FEV 1** | **Thoracic/abdominal surgery** | **Yes**  **β(95%CI) *P*-value** | **No**  **β(95%CI) *P*-value** | **Total**  **β(95%CI) *P*-value** |
|  | **Model I** |  |  | P-interaction: 0.514 |
|  | A straight-line effect | -1.35 (-2.96, 0.27) 0.1020 | -1.09 (-1.89, -0.29) 0.0075 | -1.11 (-1.82, -0.39) 0.0025 |
|  | **Model II** |  |  | P-interaction: 0.144 |
|  | Fold points (K) | 113 | 96 | 96 |
|  | < K-segment effect 1 | -6.67 (-12.03, -1.31) 0.0150 | 2.50 (-3.15, 8.14) 0.3859 | 0.86 (-4.36, 6.07) 0.7474 |
|  | >K-segment Effect 2 | -0.05 (-2.09, 1.98) 0.9586 | -1.29 (-2.14, -0.43) 0.0032 | -1.21 (-1.98, -0.44) 0.0020 |
|  | Effect size difference of 2 versus 1 | 6.62 (0.26, 12.97) 0.0416 | -3.78 (-9.68, 2.11) 0.2083 | -2.07 (-7.50, 3.37) 0.4564 |
|  | Equation predicted values at break points | 2853.52 (2755.94, 2951.11) | 3369.74 (3313.44, 3426.05) | 3310.64 (3259.56, 3361.73) |
|  | Log likelihood ratio tests | 0.037 | 0.206 | 0.455 |

Abbreviations: FVC: forced vital capacity; FEV1, forced expiratory volume in one second. Weighted by: full sample mobile examination center exam weight. Outcome variable: baseline FVC, baseline FEV 1. Exposure variable: lactate dehydrogenase. Adjusted for age, gender, race/Hispanic origin, education level, thoracic/abdominal surgery, respiratory disease, cigarette, weight, standing height, systolic blood pressure, diastolic blood pressure, glucose, serum, albumin, globulin, cholesterol, creatinine, alanine aminotransferase. When P<0.05 in Model I, the model showed a straight-line effect. When P>0.05 in Model I, the model showed a segmented effect in Model II, with the K value being the lactate dehydrogenase level at the fold point; β represents the slope of the curve, β for segments with P<0.05 was statistically significant. The K value is the inflection point, which is the level of lactate dehydrogenase content at which the relationship between lactate dehydrogenase and lung function changes.
